# Approaches for integrating heterogeneous RNA-seq data reveal cross-talk between microbes and genes in asthmatic patients

**DOI:** 10.1186/s13059-020-02033-z

**Published:** 2020-06-22

**Authors:** Daniel Spakowicz, Shaoke Lou, Brian Barron, Jose L. Gomez, Tianxiao Li, Qing Liu, Nicole Grant, Xiting Yan, Rebecca Hoyd, George Weinstock, Geoffrey L. Chupp, Mark Gerstein

**Affiliations:** 1 Program in Computational Biology and Bioinformatics, Yale University, New Haven, CT USA; 2 The Jackson Laboratory for Genomic Medicine, Farmington, CT USA; 3 Division of Medical Oncology, Ohio State University College of Medicine, Columbus, OH USA; 4 Department of Biomedical Informatics, Ohio State University College of Medicine, Columbus, OH USA; 5 Section of Pulmonary, Critical Care, and Sleep Medicine, Department of Internal Medicine, Yale University School of Medicine, New Haven, CT USA; 6 Department of Molecular Biophysics and Biochemistry, Yale University, New Haven, CT USA; 7 Department of Computer Science, Yale University, New Haven, CT USA; 8 Department of Statistics and Data Science, Yale University, New Haven, CT USA

## Abstract

Sputum induction is a non-invasive method to evaluate the airway environment, particularly for asthma. RNA sequencing (RNA-seq) of sputum samples can be challenging to interpret due to the complex and heterogeneous mixtures of human cells and exogenous (microbial) material. In this study, we develop a pipeline that integrates dimensionality reduction and statistical modeling to grapple with the heterogeneity. LDA(Latent Dirichlet allocation)-link connects microbes to genes using reduced-dimensionality LDA topics. We validate our method with single-cell RNA-seq and microscopy and then apply it to the sputum of asthmatic patients to find known and novel relationships between microbes and genes.

## Introduction

### Linking high-dimensional, heterogeneous datasets

**Fig. 1:**
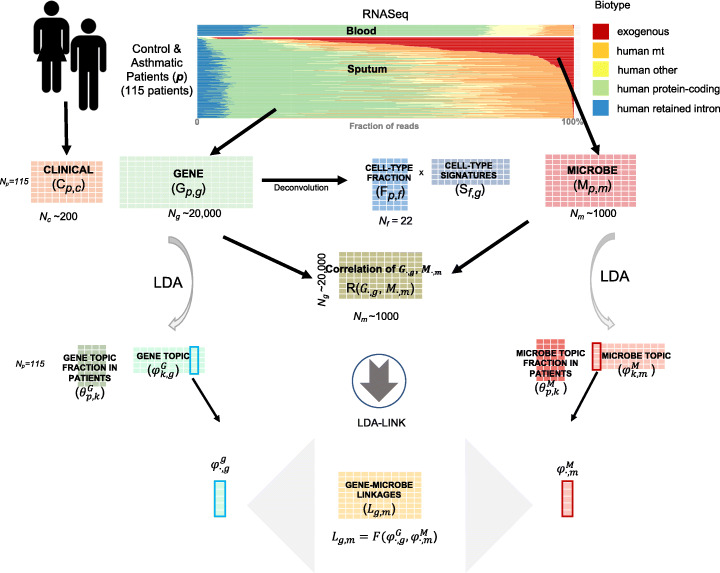
Overview of the analysis approach. The RNA-seq alignment summary for control and asthmatic sputum shows highly variable fractions of reads that aligned to different biotypes, relative to the blood. Alignments to the protein-coding biotype were used to generate the gene expression table (**G**), which was then deconvolved into a cell-type fraction (***F***) and cell-type signature (**S**) tables. The exogenous reads were used to generate the microbe table (***M***). These matrices were then related to the clinical data table (**C**) and to each other, e.g., correlation of a gene and microbe across all patients as ***R*(*G***_***.,g***_**,*M***_***.,m***_**)**. In addition, latent Dirichlet allocation (LDA) was used to de-noise tables ***G*** and ***M***, and then LDA-link was used to link genes to microbes (***L***) using the LDA topic distributions (***F***(φ^***G***^***.,g***, φ^***M***^***.,m***)

RNA sequencing (RNA-seq) has become a standard method of analyzing complex communities. Depending on the sample type, these data can be very heterogeneous. A key problem tackled in this paper is dealing with the heterogeneity and noise in RNA-seq data in samples such as sputum. This can be appreciated by comparing sputum RNA-seq to a more traditional experiment, e.g., blood RNA-seq, where the sample can be collected consistently and that contains relatively well-defined cell types (Fig. 1). In the blood, the vast majority of RNA-seq reads align to the human genome, and the goal is often to relate the expression of the genes to a phenotype. By contrast, sputum may be less consistently collected, its cell types are less well-defined, and it may contain RNA from microbes and other organisms that act as cryptic indicators of the environment. But within this complexity is an opportunity—the interactions between immune cells and microbes may provide clinically meaningful information from a non-invasive sample. Moreover, generating RNA-seq data from the sputum can be technically easier than other methods to study microbe and immune cell interactions, such as by culturing sputum microbes or by flow sorting sputum immune cells. Here, we present a strategy for dealing with the complexity that uses a number of supervised and unsupervised techniques such as cell-type signatures and latent Dirichlet allocation (LDA). These techniques can produce a low-dimensional representation of common groups of genes, microbes, or other features that tend to increase or decrease in abundance together. Our approach is useful when the heterogeneity comes from the sample type (e.g., sputum) and especially when the samples derive from a heterogeneous population of individuals, such as patients with asthma.

### Interactions between the host and microbes in the lung

Asthma is a disease of the airway that can present with diverse clinical phenotypes. Much work has focused on identifying subgroups of the disease and how each subgroup responds to treatment. For example, Yan et al. introduced transcriptional endotypes of asthma, and the Severe Asthma Research Program defined five subtypes of asthma [1, 2]. Some of these subgroups respond differently to environmental and microbial triggers, such as fungal spores. Some fungi have well-defined effects on asthma, but the role of many microbes remains uncertain. A simplified model assigns microbes to one of three categories: pathogenic organisms that cause inflammation, beneficial organisms that reduce inflammation, and commensal organisms that have no effect on inflammation. The majority of the organisms in the lungs are expected to have no effect, and severe asthmatics are expected to have more pathogenic and fewer beneficial microbes. However, the reality is more complex; organisms can interact with the lung in myriad ways, such as through innate responses to pathogens, small-molecule interactions, and others [3, 4].

### Inferring immune cell fractions from RNA-seq data

The pathology of microbes is often inferred by the number and type of immune cells observed in samples, such as sputum total leukocyte counts [5, 6]. A standard method for counting immune cells in sputum samples uses microscopy, but the resolution is limited to a few cell types [7]. Other cell-counting methods such as flow sorting can be challenging because of the viscosity and highly variable cell numbers in sputum. An alternative strategy uses cell type-specific expression patterns to deconvolve RNA-seq reads from mixtures of cells into fractions of different immune cells [8]. This deconvolution also effectively de-noises heterogeneous datasets by greatly reducing the number of dimensions. Importantly, the RNA needed for this analysis can be purified without poly-A enrichment—here, we use human ribosomal RNA depletion—which allows for the simultaneous analysis of microbial and human transcripts.

### Supervised deconvolution and the microbiome

While deconvolution to cell fractions effectively de-noises human RNA-seq data, an equivalent supervised dimensionality reduction method does not exist for microbes. One can reduce the dimensions by collapsing microbial strains to different taxonomic ranks (e.g., genus or family); however, taxonomy is notoriously imprecise at defining behavior. For example, many bacteria in the genus *Escherichia* are human commensals, whereas *Escherichia coli* OH157:H7 causes hemorrhagic colitis. Alternatively, one can group sequences by the metabolic pathways observed, although this requires high-depth sequencing. Here, we propose a method to reduce the dimensionality of microbes by first linking the microbes to human genes and then applying supervised gene dimensionality-reduction methods (e.g., deconvolution to cell types).

### Unsupervised decomposition including LDA

A number of algorithms have been developed to infer higher-order structures within high-dimensional datasets. Latent Dirichlet allocation (LDA) was first used in text mining applications to learn the set of topics in documents and has since been used in a broad range of applications including marketing, genomics, drug-pathway relationships, and others [9–12]. Unlike some other unsupervised decomposition methods (e.g., principal component analysis (PCA), non-negative matrix factorization (NMF)), the result is not a linear decomposition. The topic distributions from LDA are governed by a series of hyper-parameters that define distributions from which the “words” (in this case, genes or microbes) are drawn. This non-linear character has advantages in complex, sparse, or noisy datasets and RNA-seq such as sputum RNA-seq or single-cell RNA-seq.

In this paper, we use RNA-seq of sputum samples from asthmatic patients to demonstrate dimensionality-reduction strategies and identify microbe-host relationships. We map RNA-seq reads onto human or microbial genomes and relate the resulting abundance matrices to each other and to clinical data. Further, we deconvolve the human reads into fractions of the various cell types that make up the sputum. Finally, we relate the human genes and microbes using a method we call the LDA-link, which identifies relationships between genes, microbes, and cell types. These methods represent a general strategy for dealing with heterogeneous RNA-seq data that is applicable to other sample types beyond sputum.

## Results

### Sequencing and processing with the extracellular RNA processing toolkit (exceRpt) pipeline

We collected induced sputum samples from 115 patients with heterogeneous asthma phenotypes and sequenced these samples using RNA-seq. The median read depth per sample was 47.5 million, which meets the depth recommendations for analyses of this type [13]. We processed these reads through the exceRpt pipeline [14], which conservatively matches reads to genomes in a sequential order designed to reduce experimental artifacts. In brief, we first aligned the quality-filtered reads to the UniVec database of common laboratory contaminants [3] and then aligned the remaining reads to human ribosomal sequences before aligning them to the human genome. We excluded samples with a low ratio of transcript alignments to intergenic sequence alignments and then aligned the remaining reads to the comparably large sequence space of non-human genomes. We first aligned reads to the relatively well-curated ribosomal databases of bacteria, fungi, and archaea (e.g., Ribosomal Database Project [4]) and then to curated genomes of bacteria, fungi, viruses, plants, and animals. The percent of reads mapping to different biotypes was highly heterogeneous; a median of 60% of the reads aligned to the human reference genome and 50% to annotated transcripts (Fig. 1, green bars). A median of 0.7% of the input reads aligned to exogenous sources, with some samples containing as much as 28.1% exogenous reads. As a control, we applied the same protocol to blood samples, which demonstrated more homogeneity than sputum (Fig. 1, top, “blood”).

### Overview of the analysis approach

The goal of the analysis was to infer meaningful relationships between the numbers and origins of the RNA-seq reads and relate them to clinical phenotypes. We conceptualized the clinical information and RNA-seq alignments as a series of tables (Fig. 1). The clinical table includes patient data collected at the clinic, ***C***, including age, weight, and lung function tests, with rows indexed by patient (*N*_*p*_ = 115) and roughly 200 clinical variables (*N*_*c*_). Alignments to human protein-coding regions created the gene table, ***G***, with *N*_*p*_ rows, as above, and roughly 20,000 genes (*N*_*g*_). Alignments to exogenous genomes created the microbe table (***M***) with *N*_*p*_ rows and roughly 1000 microbes (*N*_*m*_). Given these three tables (***C***, ***G***, and ***M***), the basic analysis framework is to correlate columns or rows within or between tables. We represent this by a matrix of correlations, **R**(***X***_***∙*,*i****,*_***Y***_***∙*,*j***_), where ***X***_***∙*,*i***_ is the *i*th column of table ***X*** and ***X***_***∙*,*j***_ is the *j*th column of table ***Y***. This correlation is summed over the other index, usually *p*. For example, we test the relationship between age and the abundance of each microbe **R**(***C***_**∙,age**_, ***M***_***∙*,*m***_) across all patients. Similarly, we correlate the expression of a gene (e.g., *TLR4)* with microbe (e.g., *Candida*) **R**(***G***_***∙*,*TLR*4**_, ***M***_***∙*,*Candida***_).

Individual correlations can be difficult to interpret, particularly in heterogeneous, sparse, or noisy datasets. Organizing the genes into relevant pathways or cell types can reduce the dimensionality and de-noise the analysis. To this end, we deconvolved the gene table (***G***, *N*_*p*_*by N*_*g*_) into a cell-type fraction table (***F***, *N*_*p*_*by N*_*f*_) and a cell-type signature table (***S***, *N*_*f*_*by N*_*g*_). However, an analogous supervised method does not exist for the microbes. Therefore, we applied an unsupervised dimensionality-reduction approach, latent Dirichlet allocation (LDA), which provides topic distributions in patients (*θ*^*G*^, *N*_*p*_*by N*_*k*_) across a smaller number (*k* = 10) of gene topic (*φ*^*G*^, *N*_*k*_*by N*_*g*_). This can also be done to the microbe table M and get ***θ***^*M*^ and ***φ***^*M*^, and the gene and microbe topic can be correlated (e.g., **R(**${\boldsymbol{\theta}}_{\bullet, \boldsymbol{g}}^{\boldsymbol{G}}$, ${\boldsymbol{\theta}}_{\bullet, \boldsymbol{m}}^{\boldsymbol{M}}$**)** over all patients).

The framework described above is useful for identifying linear relationships, but non-linear relationships are also possible. For example, a microbe sensed by a human immune cell could lead to the activation of a transcription factor and the expression of several genes, each of which would have a non-linear relationship to microbe abundance. To identify such relationships, we applied a non-linear ensemble learning algorithm [15, 16], using the de-noised inputs for each gene and microbe (***φ***^*G*^ and ***φ***^*M*^). We call this method the LDA-link. Further, we relate the gene and microbe links identified to cell fractions and thereby relate how the host is responding to microbes with regard to immune cell type response with a particular gene.

### Analysis of human-aligned reads

**Fig. 2:**
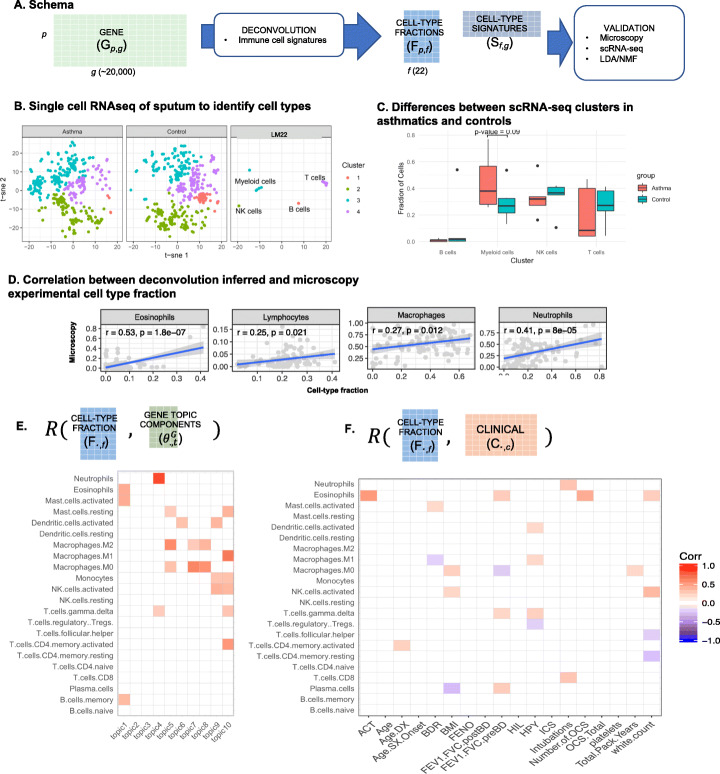
Deconvolution of RNA-seq human reads into cell-type fractions using cell-type signatures. **a** Schematic showing the estimation of a cell-type fraction table and validation steps. **b** Cell-type fractions were validated using single-cell RNA-seq. Sputum from asthmatics and controls were clustered with reference cell-type expression to label clusters. **c** The abundances of scRNA-seq cell-type fractions are highly variable between individuals. **d** Correlation between the deconvolved cell-type fraction table and cell counts by microscopy. **e** Pearson correlation of the cell-type fraction table, ***F***, and the LDA topic components. Only significant correlations after FDR correction are shown. **f** Correlation between the cell-type fraction table and the clinical table, ***C***. Only significant correlations after FDR correction are shown. ACT, asthma control test score; Age.DX, age of asthma diagnosis; Age.SX.Onset, age of symptom onset; BDR, bronchodilator response, BMI, body mass index; FENO, forced expiratory nitric oxide; FEV1.FVC.postBD, the ratio of forced expiratory volume in 1 s to the forced vital capacity after treatment with a bronchodilator; FEV1.FVC.preBD, the ratio of forced expiratory volume in 1 s to the forced vital capacity before treatment with a bronchodilator; HIL, hospitalizations in lifetime; HPY, hospitalizations per year; ICS, average daily inhaled corticosteroid use; Number.of.OCS, average number of oral corticosteroids used; OCS.Total, lifetime total oral corticosteroid use

Working toward the hypothesis that we can conceptualize human-aligned sputum RNA-seq reads as a mixture of immune cell types, each with a distinct expression profile, we deconvolved the gene table (***G***) into a table of fractions of component cells type (***F***) and cognate cell-type signatures (***S***) by solving the formula ***G*** = ***FS***. This method relies on knowing the signature gene set in each cell type, for which we used high-quality reference sets from experimentally isolated and sequenced circulating immune cells [8]. To validate that these circulating immune cells behaved similarly to those in sputum, we generated additional datasets including single-cell RNA-seq (scRNA-seq) and microscopy, and then compared the results to the deconvolution table ***F*** and to unsupervised decomposition (Fig. 2a, schema).

#### Evaluation of deconvolution results by scRNA-seq

First, we performed scRNA-seq on the sputum of a cohort of similar patients (five control and five asthmatics). The single-cell sequences clustered into four groups (Fig. 2b, first and second panels). In order to identify the clusters, we co-clustered the reference cell types with the scRNA-seq data (Fig. 2b, third panel); the reference cells grouped by lineage each with a different scRNA-cluster. For example, the reference cells of lymphoid origin (e.g., T cells, NK cells) clustered together with one of the scRNA-seq clusters, and the reference cells of myeloblast origin (e.g., neutrophils, eosinophils, macrophages) clustered together and with another scRNA-seq cluster. In aggregate, the asthmatic group had fewer B cells and more myeloid cells (Fisher’s exact test, *p* values 2e−8 and 7e−7 for B cells and myeloid cells, respectively). However, the number of cells in each cluster was highly variable between individuals; for example, nearly all of the B cell cluster was found in one individual (Fig. 2c, control B cell outlier). For these five patients, the number of cells in the myeloid lineage cluster showed the most distinct separation between asthmatics and controls but was not significant (Mann-Whitney-Wilcoxon signed-rank test, *p* value = 0.09) (Fig. 2c). From this analysis, we concluded that (1) the blood-derived cell profiles appropriately fit the sputum cell types, (2) no additional cell types were needed to deconvolve the sputum bulk RNA-seq data, and (3) the high variability would require larger sample sizes to detect differences between asthmatics and controls.

#### Evaluation of deconvolution results by microscopy

Second, we evaluated a random subset (~ 50%) of the samples by microscopy and manually counted the number of neutrophils, eosinophils, lymphocytes, and macrophages. We found good agreement with ***F***, when cell counts could be directly compared, i.e., neutrophils and eosinophils were both present in ***F*** and counted by microscopy (*F* test *p* values = 8e−5 and 1.8e−7 for neutrophils and eosinophils, respectively). In cases where the deconvolution method gave higher resolution (e.g., M0, M1, and M2 macrophages versus one type of macrophage by microscopy), the aggregation of the relevant columns in ***F*** correlated well with the microscopy counts (*F* test *p* values = 0.012 and 0.021 for macrophages and lymphocytes, respectively) (Fig. 2d).

#### Association of cell fractions with clinical features

Having validated the deconvolution of sputum samples (table ***F***), we then correlated the cell fractions with clinical features (**R**(***F***_***∙*,*f***_, ***C***_***∙*,*c***_) for all patients). We found that the changes in fractions of several cell types were highly correlated with clinical features (Fig. 2e). For example, the fraction of T-regulatory cells negatively correlated with the number of hospitalizations per year (*p* value = 0.002, all correlations false discovery rate corrected, see the “Methods” section).

#### Evaluation of deconvolution results by unsupervised decomposition

We compared the signal captured by cell-type deconvolution to an unsupervised decomposition method: LDA. Using LDA, we factored the gene expression table into ten topics that conceptually represent gene expression programs. This resulted in a gene-topic-fraction-in-patient table (***θ***^*G*^*, N*_*p*_*by N*_*k*_) with *N*_*k*_ = 10 topics, as well as the corresponding gene-topic table (***φ***^*G*^ [*N*_*k*_*by N*_*g*_]), that is analogous to the supervised deconvolution tables ***F*** and ***S***. We correlated the cell-type fraction table with the gene topics fraction table (**R**(***F***_***∙*,*f***_, ***θ***_***∙*,*k***_) for all patients and found agreement between LDA and the cell signature-based deconvolution for the most prominent cell type, neutrophils (*p* value = 1.7e−34) (Fig. 2d, topic 4). Gene set enrichment analysis of the genes in this topic showed enrichment in the neutrophil chemotaxis pathway (Additional file 1**:** Fig. S8 B). The top genes in each topic can be found in Additional file 1: Fig. S7 and the complete membership in Additional file 2: Table S1.

However, the remaining topics comprised multiple cell types. This suggests that LDA can identify distinct but partially overlapping features in ***G***. According to the clustering of ***θ***^*G*^, a subgroup of severely asthmatic patients associated with topic 4 (Additional file 1: Fig. S8A). The top-weighted genes in topic 4 were enriched for the pathways “neutrophil chemotaxis” and “asthma-related genes” (Additional file 1: Fig. S8B). These pathways were not enriched in the analogous cell-type signature table ***S***, suggesting that LDA topics are distinct from the cell-type signatures, but are also clinically relevant. Moreover, the top-weighted genes in topic 1 of the gene topic component table were mitochondrial genes, and topic 1 was strongly correlated with age. This link shows strong support in the literature, as reactive oxygen species produced by the mitochondria reduce their function over time [17]; however, we did not observe this relationship for any cells in the cell-type fraction table (***F***). Another method using a very different algorithm than LDA, non-negative matrix factorization (NMF), showed strong agreement with LDA (*p* value < 2.2e−16) (Additional file 1: Fig. S2, Nmf.1). This supports the use of supervised deconvolution methods for identifying signals that are different than those from unsupervised methods. Unsupervised decomposition should be considered a distinct set of features from those found through deconvolution.

### Analysis of exogenous reads

After filtering out contaminants and human reads, we assembled the set of reads that aligned to exogenous genomes into a microbe table (***M***). The exogenous sequences aligned to mostly bacteria and fungi, although we also observed a few arthropod and helminth reads. The dominant phyla observed were from the bacterial kingdom: Proteobacteria, Firmicutes, and then Bacteroidetes. The abundance of Proteobacteria is in contrast to observations from the gut where Bacteroidetes predominate [18]. Also notable was the presence of two phyla of fungi among the eight most abundant overall, although this was in lower abundance than many of the bacterial phyla.

#### Microbe correlations with clinical information and cell fractions

**Fig. 3:**
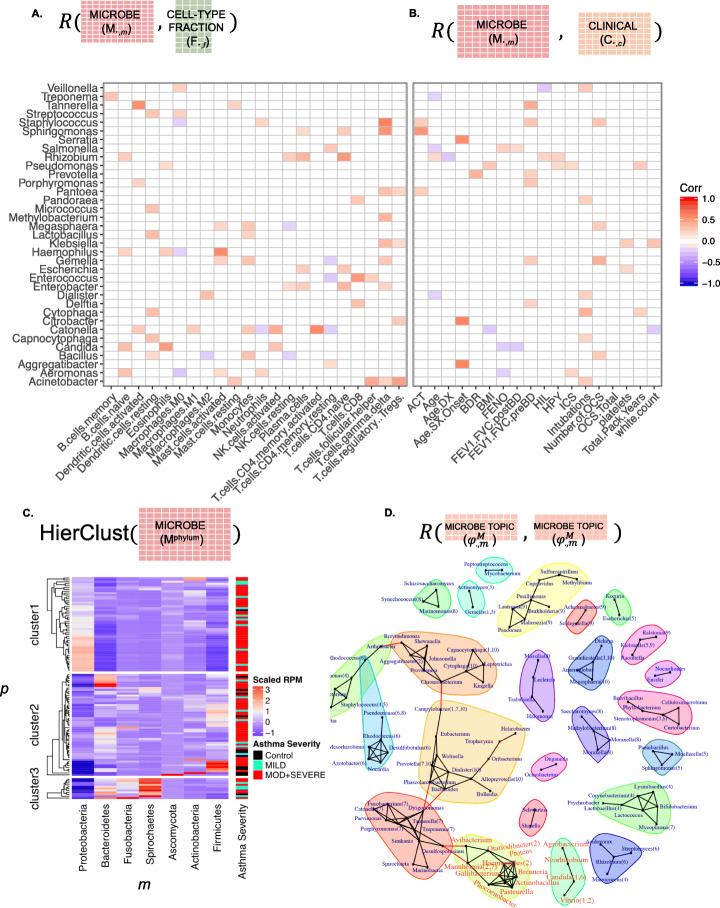
Exogenous RNA-seq analysis. **a** Correlations between microbe relative abundances and the cell-type fraction table. **b** Correlation between microbe abundance and clinical information. Only significant correlations after FDR correction are shown. ACT, asthma control test score; Age.DX, age of asthma diagnosis; Age.SX.Onset, age of symptom onset; BDR, bronchodilator response; BMI, body mass index; FENO, forced expiratory nitric oxide; FEV1.FVC.postBD, the ratio of forced expiratory volume in 1 s to the forced vital capacity after treatment with a bronchodilator; FEV1.FVC.preBD, the ratio of forced expiratory volume in 1 s to the forced vital capacity before treatment with a bronchodilator; HIL, hospitalizations in lifetime; HPY, hospitalizations per year; ICS, average daily inhaled corticosteroid use; Number.of.OCS, average number of oral corticosteroids used; OCS.Total, lifetime total oral corticosteroid use. **c** Hierarchical clustering by phylum relative abundances shows a cluster enriched in severe asthmatics, driven by high abundances of Proteobacteria. **d** A co-abundance network of microbes with an overlay of LDA when network modules correlate with topic membership

We correlated the microbe abundances to clinical information (***R***(***M***_***∙*,*m***_, ***C***_***∙*,*c***_) for all patients) (Fig. 3a). *Klebsiella* was associated with increased total white blood cell counts (*p* value = 0.001), as has been described previously [19]. *Candida* was associated with worse lung function test results (e.g., forced expiratory volume and forced vital capacity (*p* value = 0.02)).

We next correlated microbe abundances to human immune cell fractions (**R**(***M***_∙, ***m***_, ***F***_∙, ***f***_) for all patients) (Fig. 3b). Several correlations demonstrated results with strong literature precedence. For example, studies have previously shown that *Haemophilus* associates with eosinophilia [20], and we observed a significant correlation between *Haemophilus* and the fraction of eosinophils (*p* value = 0.005). We also observed a significant correlation between *Haemophilus* and activated mast cells (*p* value = 9e−12), suggesting an alternative route to *Haemophilus*-induced inflammation [21]. Moreover, the fungal genus *Candida* was also significantly correlated with eosinophils (*p* value = 1e−8), even more strongly than *Haemophilus*.

#### Dimensionality reduction for microbes: clustering and networks

We attempted to de-noise the microbe table (***M***) with a variety of dimensionality-reduction techniques. First, we collapsed the microbes by taxonomy, grouping them to the rank of phylum (***M***^**phylum**^), and then hierarchically cluster the patients based on their phylum abundance (Fig. 3c, **HierClust**(***M***^**phylum**^)). The hierarchical clustering showed that the phylum distributions formed three clusters of patients. We related these clusters to the clinical variable “asthma severity,” defined by the amount of fluticasone or equivalent per day used to control symptoms (mild, < 200 μg; moderate, 200–800 μg; severe, > 800 μg). We observed that one of the clusters was enriched for patients identified as having moderate or severe asthma (Fisher’s exact test, *p* value = 0.005). This cluster was characterized by the highest relative abundance of the phylum Proteobacteria (Fig. 3c). Notably, the genus *Haemophilus* belongs to this phylum, consistent with the correlations observed at the genus rank (Fig. 3a, b).

Similarly, we could de-noise the microbe table using a co-abundance network, by correlating the genus-level abundances (**R**(***M***_∙, ***m***_, ***M***_∙, ***m***_) and identifying significant modules (Additional file 1: Fig. S5). An interpretation of these modules is that they define metabolic niches, where microbes either directly compete for metabolites or there is interdependency in metabolite production. Such networks could be created from other tables, such as the topic distribution of microbes  for all the topics (Fig. 3d). These modules represent another unit that could be related to the clinical information (***C***) and the cell-type fractions (***F***).

### LDA-link for the identification of links between genes and microbes

How much cross-talk exists between the airway microbes and human cells remains contentious [22]. We feel this is partly due to the heterogeneous and noisy data from airway samples, where it is often challenging to find strong correlations using standard algorithms. We therefore sought to link genes to microbes via a method we dubbed the LDA-link.

As is often the case, these RNA-seq data lack a gold standard set of labels for the links between genes and microbes that can train a machine learning algorithm. Therefore, the LDA-link uses a strategy similar to self-supervised methods, which create a pseudo-gold standard, train a supervised model using the pseudo-gold standard, and then apply the model to the remainder of the data [23–30]. In this case, the training set was defined by a small number of very strong linear correlations and an equal number of very low correlations.

**Fig. 4:**
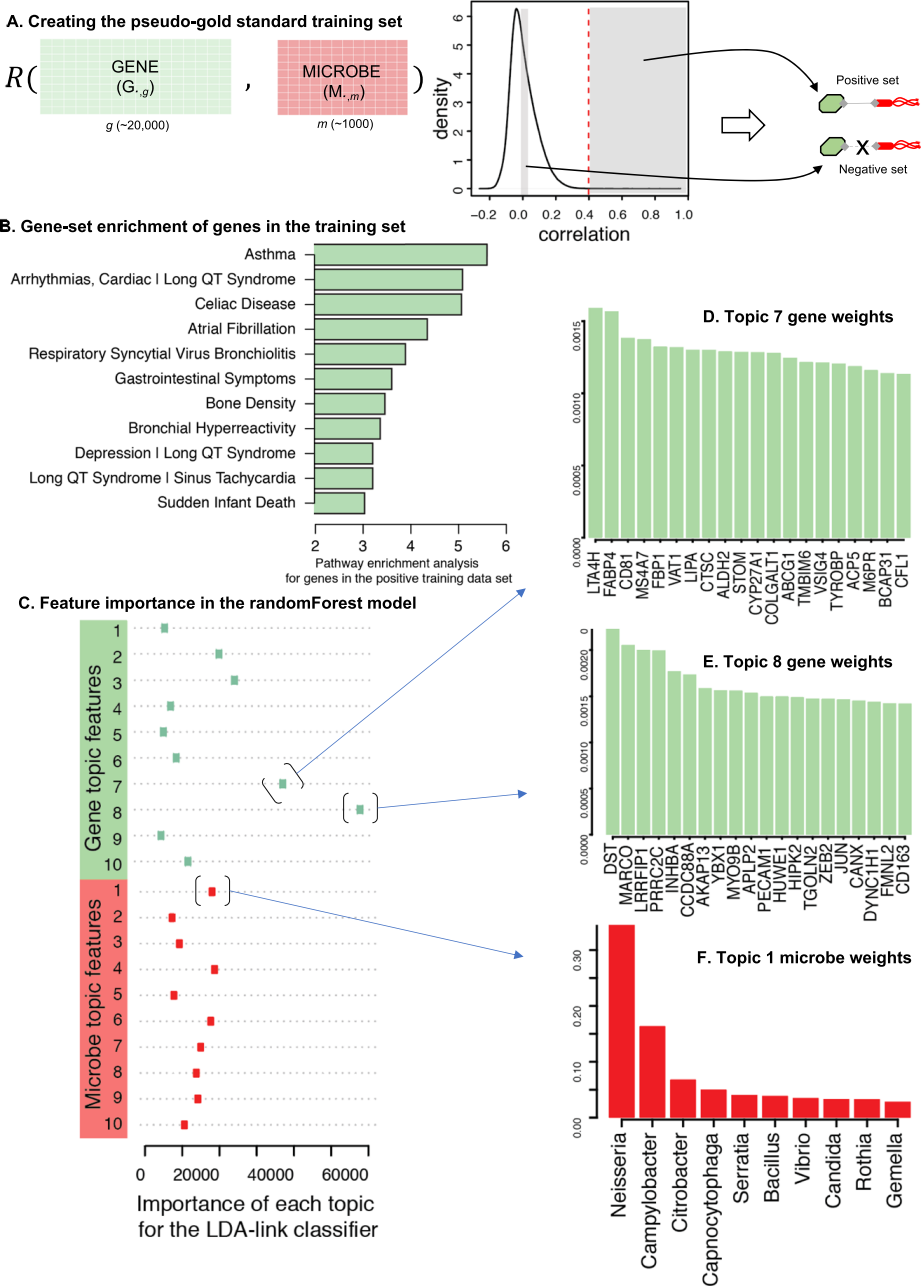
Method of LDA-link for predicting links between genes and microbes. **a** The method for identifying a pseudo-gold standard training dataset through identifying a set of very strong linear correlations and an equal number of very low correlations. **b** Gene set enrichment of the pseudo-gold standard genes shows enrichment in pathways related to asthma. The *x*-axis is the -log(*p* value). **c** The importance of features (LDA topics for genes and microbes) in the randomForest model by Gini index. The top 20 associated gene in topic 8 (**d**) and topic 7 (**e**) of genes, and topic 1 (**f**) of microbes

Specifically, we first related columns between the gene and microbe tables (**R**(*G*_∙, *g*_, *M*_∙, *m*_)), yielding many low-scoring correlations. A relatively small number of the correlations were strong (**R** > 0.4) and highly significant (*p* < 1e−5 and FDR < 0.016) (Fig. 4a). We selected these strong correlations as true-positive links between genes and microbes in the self-supervised training set and non-correlated pairs (− 0.05 < **R** < 0.05) as the true-negative links. We tested the validity of this true-positive label by gene set enrichment analysis. The genes involved in these strong correlations were enriched for pathways related to microbial interactions in the airway, including “asthma and bronchial hypersensitivity” and “respiratory syncytial virus bronchiolitis” (Fig. 4b), suggesting that the small set of strong linear correlations was relevant to asthma. These gene-microbe links were then used to train a random forest classifier.

An important idea for the method is that the input features for the random forest classifier were the unsupervised LDA topic distributions for each gene and microbe (${\varphi}_{\bullet, g}^G$*,*${\varphi}_{\bullet, m}^M$), rather than the “direct” gene counts and microbe abundances that were used to identify the linear correlations. That is, for each gene-microbe pair, we concatenated the gene and microbe topics into a single vector (length 20) that were the features used by the random forest classifier. The most important features in defining links between genes and microbes in the training set were gene topics 7 and 8 and microbe topic 1 (Fig. 4c–f). The genes that comprise the most influential gene topic 8 are enriched for the pathway “inflammatory response,” and specifically the cytokines IL2 and IL6.

We applied the trained model to all gene-microbe pairs (excluding the training set), using the LDA topic distributions as inputs. The confidence scores of the machine learning approach were strongly bimodal. Whereas the linear correlation showed many weak to mid-strength and a few strong interactions, the LDA-link resulted in many low-confidence interactions, few mid-strength, and a larger number of high-confidence interactions. Altogether, we identified 1883 high-confidence (probability > 0.95) gene-microbe interaction pairs, as opposed to the linear correlations, which yielded 862 (*p* value < 1e−5).

### Cross-talk between genes and microbes defined by LDA-link

**Fig. 5:**
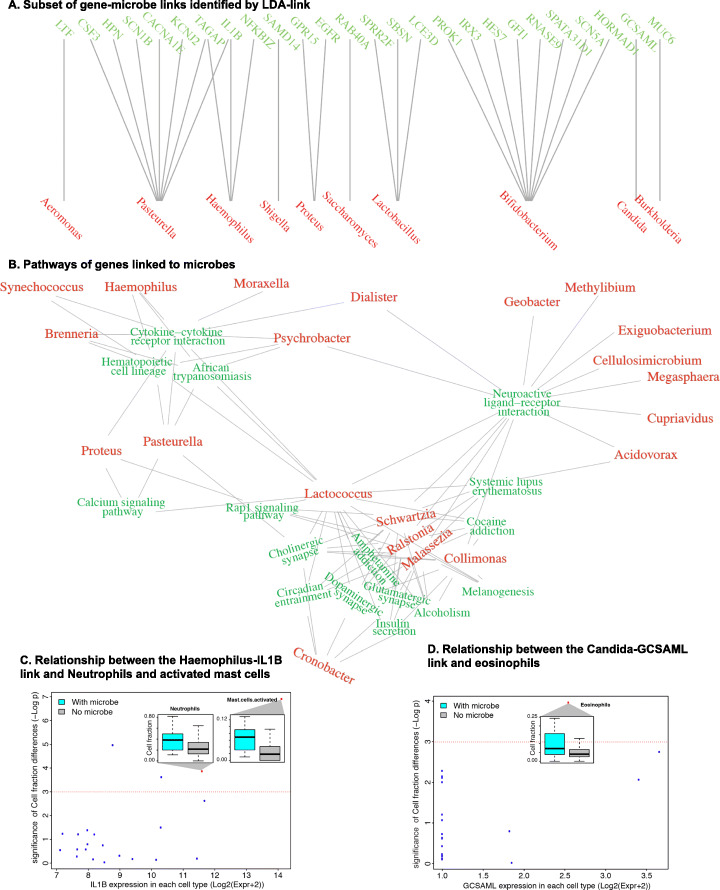
Summary of links between genes and microbes and their relationship to cell types. **a** Representative links between microbes and genes. **b** Pathway analysis of the genes found liked to microbes. **c** Example links between genes and microbes showing both gene expression in each reference cell type and the difference in abundance of the cell in samples where the linked microbe was observed

LDA-link identified connections between genes and microbes reported elsewhere in the literature as well as novel observations. A bipartite graph summarizing a subset of the connections between genes and microbes shows that in most cases, several genes are linked to each microbe (Fig. 5a, for a complete list, see Additional file 3: Table S2). Several of the observed links showed strong literature precedence. For example, the gene lactotransferrin was linked to *Aeromonas* [31], *Burkholderia* was linked to gene MUC6 [32], *Haemophilus* was linked to NFKB Inhibitor Zeta [33], and *Pasteurella* was linked to IL1B [34]. To the best of our knowledge, several novel links were also found, such as between *Haemophilus* and IL1B and between *Candida* and GCSAML. In addition to gene-microbe pairs, we layered on the pathway and cell deconvolution data to identify larger-scale effects of microbes.

Microbes were linked to genes that are enriched in pathways relating to autoimmunity and inflammation as well as cytokine receptors and their interactions (Fig. 5b). The microbes associated with cytokine pathways included *Synechococcus*, *Lactococcus*, *Dialister*, *Psychrobacter*, *Moraxella*, *Brenneria*, *Proteus*, *Haemophilus*, and *Pasteurella*. In addition, we related the cell-type signature table (***S***_*f,g*_) to identify the immune cell types that are related to each microbe (Fig. 5c). We observed the *Haemophilus*-IL1B link in monocytes and mast cells. Samples containing *Haemophilus* correlated with more activated mast cells (Spearman correlation, *p* value = 9−12) (Fig. 5c, inset) [35–38]. Similarly, the fungal genus *Candida* was linked to the gene GCSAML, which was highly expressed by eosinophils. The presence of *Candida* was associated with increased numbers of eosinophils in the airway (Spearman correlation, *p* value = 1e−8).

## Discussion

Heterogeneity and noise are common problems in biological datasets. Heterogeneity can derive from mixtures of different cell types, such as in sputum, or from sparsity, such as in microbiome or single-cell RNA-seq data. Unsupervised methods of dimensionality reduction can effectively eliminate these issues, but suffer from decreased interpretability. That is, variables are collapsed together for reasons that are often opaque. Supervised dimensionality reduction maintains interpretability because variables are collapsed using prior knowledge, such as the genes in a pathway or the expression patterns of a cell type. Here, we describe a method we dubbed LDA-link that combines supervised and unsupervised methods to de-noise the data while retaining interpretability.

The field of asthma is increasingly appreciating the role of the airway microbiome in disease. Commensal microbiota have been shown in other contexts to be strong regulators of host immune system development and homeostasis [39]. Disturbances in the composition of commensal bacteria can result in imbalanced immune responses and affect an individual’s susceptibility to various diseases, including those that are inflammatory (e.g., inflammatory bowel disease and colon cancer), autoimmune (e.g., celiac disease and arthritis), allergic (e.g., asthma and atopy), and metabolic (e.g., diabetes, obesity, and metabolic syndrome) (reviewed in [40]). Investigating the microbiota in the lower respiratory tract is a relatively new field in comparison with the extensive work on the intestinal tract. In fact, the lung was excluded from the original Human Microbiome Project because it was not thought to have a stable resident microbiome [18]. Since this initial lag, reports have defined commensal microbes within healthy individuals and identified consistently enriched or depleted taxa in such conditions as asthma, chronic obstructive pulmonary disease, and cystic fibrosis, to name a few [5, 41–44]. LDA-link facilitates the observation of relationships between microbes and genes and links them to immune cells and their responses.

While several links imply that the presence of a microbe has triggered an inflammatory response, other interactions are also suggested. The gene lactotransferrin was linked to *Aeromonas*, which has been associated with gastroenteritis and skin infections and has been previously reported to bind lactoferrin [31]. *Burkholderia*, a gram-negative bacterial genus, is recognized as an important pathogen in the mucus-filled lungs of patients with cystic fibrosis; it was linked to gene MUC6, which encodes a secreted protein responsible for the production of mucin [32]. Classical pathogenic-type interactions included *Haemophilus* linked to NFKB inhibitor zeta, which is induced by the bacterial cell wall component lipopolysaccharide [33]. In addition, *Haemophilus* was linked to the cytokine interleukin 1 beta (IL1B), an important mediator of the inflammatory response. IL1B hypersensitivity is a hallmark of the asthma phenotype. *Pasteurella* was also linked to IL1B, and its toxin has been shown to induce the expression of IL1B [34].

A number of the links identified here have been described previously through different experimental methods. For example, a severe form of asthma has been characterized by eosinophilia [45], and pulmonary candidiasis has long been associated with allergic bronchial asthma and inflammation [46]. However, the methods used to uncover these relationships we argue are more challenging than total RNA-seq of sputum or provide less information. For example, asthmatic eosinophilia was identified by microscopic counting of cells [45], as we used to validate deconvolved cell counts (Fig. 2d). In the case of candidiasis, the observation was made by culturing [46]. By contrast from these RNA-seq data, significantly more information can be obtained, such as other cell types and other microbes. In addition, these links were not found via standard correlations. By using the LDA-link, we identified associations that could not be extracted from this heterogeneous dataset. Notably, both fungi and bacteria showed these links, further highlighting the need to evaluate more than bacteria when performing microbiome experiments in the airway.

Having gained confidence in the method by finding previously reported associations, we then turn to the novel findings. The links identified here suggest processes by which lung immune cells respond to microbes. We found that mast cells respond to *Haemophilus* and *Pasteurella* via IL1B and that eosinophils respond to *Candida* via GCSAML. While experimental validation of these links is needed, these results represent observations that would be missed by analyses that do not effectively de-noise RNA-seq data in a way that maintains the interpretability of cell types, or that analyze only human RNA-seq reads. Additional follow-up is needed to establish a causal connection between these correlations.

RNA-seq continues to be a common dataset collected in biological sciences. Increasingly, its applications include the identification of distinct entities within the data, such as microbes and immune cells. For example, the roles of both microbes and immune cells are being inferred using RNA-seq from tumor biopsies and other tissues [47, 48] where the interpretation can be challenging because of the sample (sparse, low-abundance microbes) and its preservation (e.g., RNA degradation in formalin-fixed paraffin-embedded tissues). We expect the LDA-link to be useful in this and many other such contexts.

## Methods

### Patient cohort

The patient population in this study received treatment at the Yale Center for Asthma and Airway Diseases before 2016. Included were patients classified as having mild, moderate, or severe asthma, as defined by the amount of fluticasone or equivalent per day to control symptoms (mild, > 200 μg; moderate, 200–800 μg; severe, > 800 μg). Most patients were young (median age 46), female (~ 75%), and White (60%). A single sample was used from each patient, not within 2 weeks of an exacerbation event. Control samples from 13 healthy individuals were included (Additional file 4: Table S3).

### Sample collection and sequencing

Sputum induction was performed with hypertonic saline, the mucus plugs were dissected away from the saliva, the cellular fraction was separated, and the RNA was purified as described previously [49]. Briefly, RNA was purified using the All-in-One purification kit (Norgen Biotek), and its integrity was assayed by an Agilent bioanalyzer (Agilent Technologies, Santa Clara, CA). Ribosomal depletion was performed with the RiboGone-Mammalian kit (Clontech Cat. Nos. 634846 and 634847), and cDNA was created with the SMARTer Stranded RNA-seq Kit (Cat. No. 634836). Samples were sequenced using an Illumina HiSeq 4000 with 2 × 125 bp reads, with an average of 47.5 million reads per sample.

### RNA-seq processing by exceRpt

An adapted version of the software package exceRpt [14] was used to process the sputum RNA-seq data. Briefly, RNA-seq reads were subjected to quality assessment using the FastQC software v.0.10.1 (https://www.bioinformatics.babraham.ac.uk/projects/fastqc/) both prior to and following 3′ adapter clipping. Adapters were removed using FastX v.0.0.13 (http://hannonlab.cshl.edu/fastx_toolkit/). Identical reads were counted and collapsed to a single entry, and reads containing N’s were removed. Clipped, collapsed reads were filtered through the Univec database of common laboratory contaminants and a human ribosomal database before being mapped to the human reference genome (hg19) and pre-miRNA sequences using STAR [50]. Reads that did not align were mapped against a ribosomal reference library of bacteria, fungi, and Archaea, compiled by the Ribosome Database Project [51], and then to genomes of bacteria, fungi, plants, and viruses, retrieved from GenBank [51]. In cases where RNA-seq reads aligned equally well to more than one microbe, a “last common ancestor” approach was used, and the read was assigned to the next node up the phylogenetic tree, as performed by similar algorithms [14, 52].

### Data tables notation

We use the following notation to define matrices associated with patients (*N*_*p*_ = 115), genes (*N*_*g*_ = ~ 20,000), microbes (*N*_*m*_ = ~ 1000), cell fractions (*N*_*f*_ = 22), reference cell-type signature genes (*N*_*s*_ = ~ 600), and topics (*N*_*k*_ = 10) (Fig. 1):

***C***: clinical table [*N*_*p*_*by N*_*c*_] where *c* is the clinical index

***G***: gene table [*N*_*p*_*by N*_*g*_] bulk RNA-seq table, FPKM expression of each gene for each patient

***M***: microbe abundance table [*N*_*p*_*by N*_*m*_], relative abundances of each microbe for each patient

***F***: cell fraction table [*N*_*p*_*by N*_*f*_] resulting from the deconvolution of ***G***_*p,g*_

***S′***: reference cell-type expression table [*N*_*f*_*by N*_*s*_] from the RNA-seq of experimentally isolated immune cells where *s* is the index of genes in the signature set

***S***: expanded all signature table [*N*_*f*_*by N*_*g*_] resulting from solving ***S*** = ***F***^***−1***^***G***

***θ***^*G*^: patient topic table [*N*_*p*_*by N*_*k*_] after LDA inference based on gene table ***G***_*p,g*_

***φ***^*G*^: gene topic table [*N*_*k*_*by N*_*g*_] after LDA inference based on gene table ***G***_*p,g*_

***θ***^*M*^: patient topic table [*N*_*p*_*by N*_*k*_] after LDA inference based on microbe table ***M***_*p,m*_

***φ***^*M*^: microbe topic table [*N*_*k*_*by N*_*m*_] after LDA inference based on table ***M***_*p,m*_

***L***: gene-microbe link table [*N*_*g*_*by N*_*m*_] predicted by LDA-link

### Dimensionality reduction

#### Supervised deconvolution

The gene table (***G***) was deconvolved using the transcriptomes from 22 flow cytometry-sorted and sequenced immune cell types using the CIBERSORT tool [8]. Briefly, characteristic gene expression patterns for each cell type were used to estimate the fraction of each cell type in the sample by solving for the equation:

$$ G= FS^{\prime } $$

where ***G*** is the gene table of human protein-coding gene expression from the exceRpt pipeline, ***F*** is the cell fraction table, and ***S′*** is the reference cell-type gene expression signature from experimentally isolated and sequenced cell types. Support vector regression was used to perform variable selection, reducing the number of characteristic genes used to distinguish cell types and thereby reducing overfitting. The above equation was then solved to provide an estimate of ***F***. *P* values for the fit of ***F*** to ***G*** demonstrated that all samples were significant at *α* = 0.05. Following the solution of ***F***, the cell type expression, ***S***, expanded to all the other, non-signature genes, were inferred using the same equation.

#### Justification for unsupervised decomposition using LDA

We compared LDA to several commonly used dimensionality reduction techniques. As a canonical method, principal component analysis (PCA) uses an orthogonal transformation to convert a data matrix into a set of linearly uncorrelated principal components, but it may give negative numbers. Similar to PCA, non-negative matrix factorization (NMF) can be represented by a linear combination of all the variables, but it requires all the matrices to have only non-negative elements.

LDA has two advantages over these methods that we demonstrate using a classic machine learning dataset. First, LDA provides a sparser decomposition that yields more robust and interpretable representations. By controlling the hyper-parameter *α*, LDA achieves better sparseness of low-dimensional patterns (Additional file 1: Fig. S10A). Second, another important aspect of the decomposition is that it should preserve the distances (correlations) between two variables in the original data [53]. The correlations from LDA document-to-topic distributions more closely resemble the linear correlations than the feature matrix (*W*) from NMF **(**Additional file 1: Figure S10B-C).

#### Decomposition through a generative model

The gene table, ***G***, was decomposed using LDA and NMF. For LDA, the abundance values for bulk RNA-seq and exogenous RNA were scaled down to reduce computation intensity during the Gibbs sampling. More simply, the RPM expression values were converted to integers and then divided by 10. The max value was set to 1000. Each patient represents a document, and the genes and microbes were treated like a dictionary of words in the traditional LDA application. The word was gene or microbe, and the word count represents gene expression or microbe abundances. The dictionary (or corpus) includes *V* unique words (genes or microbes), and each word has an index (*v*) in the dictionary; we built LDA models for genes and microbes, respectively.

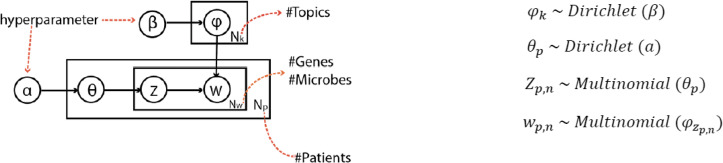



In this model, *N*_*p*_ is the number of documents, *N*_*w*_ is the number of words in a document, *N*_*k*_ is the number of topics (set as 10), and *N*_*v*_ is the total number of words in the dictionary. The variables *p*, *w*, *k*, and *v* denote indices of documents, words in a document, topics, and words in a dictionary, respectively. The vector *α*, of length *N*_*k*_, and *β*, of length *N*_*v*_, are the hyper-parameters; *θ* [*N*_*p*_ by *N*_*k*_] is the topic distribution of documents; and *φ* [*N*_*k*_ by *N*_*v*_] is the distribution of words in topics. *z* is a categorical integer between 1 and *N*_*k*_, denoting the topic of a word in a document.

The joint distribution of the LDA model is $\mathcal{P}\left(Z,W;\alpha, \beta \right)$, and *φ* and *θ* are integrated out as:

$$ \mathcal{P}\left(Z,W;\alpha, \beta \right)=\overset{\mathcal{P}\left(\mathrm{W}\right|Z,\beta \Big)\ }{\overbrace{\int_{\varphi}\prod \limits_{i=1}^{N_k}\mathcal{P}\left({\varphi}_i;\beta \right)\prod \limits_{j=1}^{N_p}\prod \limits_{t=1}^{N_w}\mathcal{P}\left({W}_{j,t}|{\varphi}_{Z_{j,t}}\right) d\varphi}}\kern0.75em \overset{\mathcal{P}\left(Z\ \right|\ \alpha\ \Big)}{\overbrace{\int_{\theta}\prod \limits_{i=1}^{N_p}\mathcal{P}\left({\theta}_i;\alpha \right)\prod \limits_{j=1}^{N_w}\mathcal{P}\left({Z}_{i,j}|{\theta}_i\right) d\theta}} $$

$$ =\prod \limits_{k=1}^{N_k}\frac{\Delta  \left({n}_{\bullet, k}+\beta \right)}{\Delta  \left(\beta \right)}\prod \limits_{s=1}^{N_p}\frac{\Delta  \left({n}_{s,\bullet }+\alpha \right)}{\Delta  \left(\alpha \right)} $$

where

$$ \Delta  \left(\alpha \right)=\frac{\prod \limits_{k=1}^K\Gamma \left({\alpha}_k\right)}{\Gamma \left(\sum \limits_{k=1}^K{\alpha}_k\right)} $$

The Gibbs sampling equation can be derived from $\mathcal{P}\left(Z,W;\alpha, \beta \right)$ to approximate the distribution of $\mathcal{P}\left(Z|W;\alpha, \beta \right)$ because $\mathcal{P}\left(\ W;\alpha, \beta \right)$ is invariant to *Z*. Given *Z*_*m*, *n*_ denotes the topic of the *n*th word in the *m*th document and also assume that its word symbol is the *v*th word in the dictionary; ¬*m*, *n* denotes all the word except the *n*th one in the *m*th document. The conditional probability can be inferred as follows:

$$ \mathcal{P}\left({Z}_{m,n}=k|{Z}_{\neg \left(m,n\right)},W;\alpha, \beta \right)=\frac{\mathcal{P}\left(Z,W;\alpha, \beta \right)}{\mathcal{P}\left({Z}_{\neg \left(m,n\right)},W;\alpha, \beta \right)}=\frac{\mathcal{P}\left(w,z\right)}{\mathcal{P}\left({w}_{m,n},\kern0.75em {w}_{\neg \left(m,n\right)},\kern0.75em {z}_{\neg \left(m,n\right)}\right)}=\frac{\mathcal{P}\left(w,z\right)}{\mathcal{P}\left({w}_{\neg \left(m,n\right)},\kern0.75em {z}_{\neg \left(m,n\right)}\right)}\bullet \frac{1}{\mathcal{P}\left({w}_{m,n}=t\right)} $$

$$ \propto \frac{\mathcal{P}\left(w,z\right)}{\mathcal{P}\left({w}_{\neg \left(m,n\right)},\kern0.75em {z}_{\neg \left(m,n\right)}\right)} $$

$$ =\frac{\prod \limits_{k=1}^{N_k}\frac{\Delta  \left({n}_{\bullet, k}+\beta \right)}{\Delta  \left(\beta \right)}\prod \limits_{p=1}^{N_p}\frac{\Delta  \left({n}_{p,\bullet }+\alpha \right)}{\Delta  \left(\alpha \right)}}{\prod \limits_{k=1}^{N_k}\frac{\Delta  \left({n}_{\neg \left(m,n\right),k}+\beta \right)}{\Delta  \left(\beta \right)}\prod \limits_{p=1}^{N_p}\frac{\Delta  \left({n}_p,\neg \left(m,n\right)+\alpha \right)}{\Delta  \left(\alpha \right)}} $$

$$ =\frac{\Delta  \left({n}_{\bullet, \kern0.5em k}+\beta \right)}{\Delta  \left({n}_{\neg \left(m,n\right),k}+\beta \right)}\bullet \frac{\Delta  \left({n}_{p,\bullet }+\alpha \right)}{\Delta  \left({n}_{p,\neg \left(m,n\right)}+\alpha \right)} $$

After Gibbs sampling, the expectation of the *θ* (doc → topic)and *φ*(topic → word) matrix can be inferred as follows given the symmetric hyper-parameters *α* and *β* were used:

$$ {\theta}_{p,k}=\kern0.5em \frac{n_{p,\bullet}^k+\upalpha}{\sum_{k\prime =1}^{N_k}{n}_{p,\bullet}^{k\prime }+{N}_k\upalpha} $$

$$ {\varphi}_{k,v}=\kern0.5em \frac{n_{\bullet, v}^k+\beta }{\sum_{v\prime =1}^{N_v}{n}_{\bullet, v\prime}^k+{N}_v\beta } $$

We instantiated the variables *θ* and *φ* to ${\theta}_{p,t}^G,{\theta}_{p,t}^M$ and ${\varphi}_{k,g}^G,{\varphi}_{k,m}^M$, respectively, where ${\theta}_{p,t}^G,{\theta}_{p,t}^M$ denotes the gene and microbe topic fraction in patient; ${\varphi}_{k,g}^G,{\varphi}_{k,m}^M$ denotes the gene and microbe topic.

### Single-cell RNA-seq

Sputum cells were separated on a Fluidigm C1 medium-sized channel. The mRNA was purified from approximately 500 pg to 1 ng of total RNA using the Clontech SMARTer Ultra Low RNA Kit and poly-dA-selected using SPRI beads and dT primers. Full-length cDNA was sheared into 200–500 bp DNA fragments by sonication (Covaris, MA, USA) and then indexed and size validated by LabChip GX. Two-nanomolar libraries were loaded onto Illumina version 3 flow cells and sequenced using 75-bp single-end sequencing on an Illumina HiSeq 2000 according to Illumina protocols. Data were cleaned, processed, aligned, and quantified following the SINCERA pipeline [54]. Briefly, the data are processed to filter genes with poor expression across all cells and cells with poor expression across most genes (≥ 5 FPKM in 2 cells of the sample, ≥ 1000 genes in a cell) and then transformed to *z*-scores. Clusters were created via principal component analysis and then t-SNE of the principal component loadings. The optimal number of clusters was identified by calculating the gap statistic (method = “Tibs2001SEmax”) using the cluster package in R.

### Pathogen-to-host correlation and link identification

Microbe relative abundances and gene TPM values were correlated as follows, with *G*_∙, *i*_ for *i* gene and *M*_∙, *j*_ for *j* microbe:

$$ R\left(i,j\right)=\frac{\sum_{p=1}^{N_p}\left({G}_{p,i}-\overline{G_{\bullet, i}}\right)\left({M}_{p,j}-\overline{M_{\bullet, j}}\right)}{\sqrt{\sum_{p=1}^{N_p}{\left({G}_{p,i}-\overline{G_{\bullet, i}}\right)}^2\ }\sqrt{\sum_{p=1}^{N_p}{\left({M}_{p,j}-\overline{M_{\bullet, j}}\right)}^2}} $$

Correlation *p* values were corrected by the method of Benjamini and Hochberg [55].

We employed a self-supervised learning framework to learn labels from the data. The links between gene and microbes that were very tightly correlated, ergo they were in one cluster, were labeled as the positive links after being confirmed by functional enrichment (gene-microbe correlations with *p* values less than 1e−5 (FDR = 0.016 and absolute correlation greater than 0.4)). Negative links in the training set were defined as an absolute correlation of less than *R*(*i*,*j*) = 0.05. Each gene and microbe could be linked to many microbes or genes, respectively.

Cross-validation was performed using a strict left-out process, as shown in Additional file 1: Figure S11. Ten percent of the training data (true-positive and negative labels) were separated as the test set, and any microbes and genes in this test set were removed from the remaining training data, leaving slightly less than 90% of the pseudo-gold standard for training. A randomForest model was trained on the remaining labels and then tested on the left-out 10%. We repeated these procedures 10 times to estimate the receiver operator characteristics of the model; the area under the curve was approximately 0.7.

### Microbe co-abundance network

The raw abundance *M* and LDA microbe topic matrices *φ*^*M*^ , which represent the microbe’s weight to each topic, were generated. The correlation network between different microbes was calculated using the Pearson correlation. The cutoff to define a co-abundance edge was 0.8 for ***R***(${\varphi}_{\bullet, m}^M$, ${\varphi}_{\bullet, m}^M$) and 0.3 for ***R***(*M*_∙, *m*_, *M*_∙, *m*_) The microbe network modules, which were densely connected to themselves but sparsely connected to other modules, were clustered based on betweenness [56] and the other algorithms; we also tested label propagation and fast greedy algorithms [57]. We also compared the LDA topics with the microbes in the same clusters. If a microbe was the top 10 most highly contributed for a topic, then we labeled the topic number in the bracket. Some microbes may have multiple topic labels because they highly contribute to multiple topics.

## Supplementary information

**Additional file 1:Figures S1-S11.** Supplemental Figures.

**Additional file 2:Table S1.** Gene-topic membership. A tab-delimited text file showing the complete topic distributions for all genes.

**Additional file 3:Table S2.** Gene-microbe links. A tab-delimited text file showing all genes-microbe pairs and the confidence of their link.

**Additional file 4:Table S3.** Patient characteristics. A Microsoft Excel spreadsheet providing summary statistics for the patients included in the study.

**Additional file 5:** Review history.

## Data Availability

Sputum bulk and single-cell RNA-seq data are available through dbGaP under the NCBI BioProject PRJNA611097 [58]. Code for LDA-link and to regenerate all figures is available at https://github.com/gersteinlab/decoasthma [59]. The code is open-source under the MIT License in compliance with the open-source initiative.
