# Supplementary material for: Approaches for integrating heterogeneous RNA-seq data reveal cross-talk between microbes and genes in asthmatic patients
Source: Genome Biol. 2020 Jun 22;21:150. doi: 10.1186/s13059-020-02033-z (PMC7310008; doi:10.1186/s13059-020-02033-z)

Supplementary information

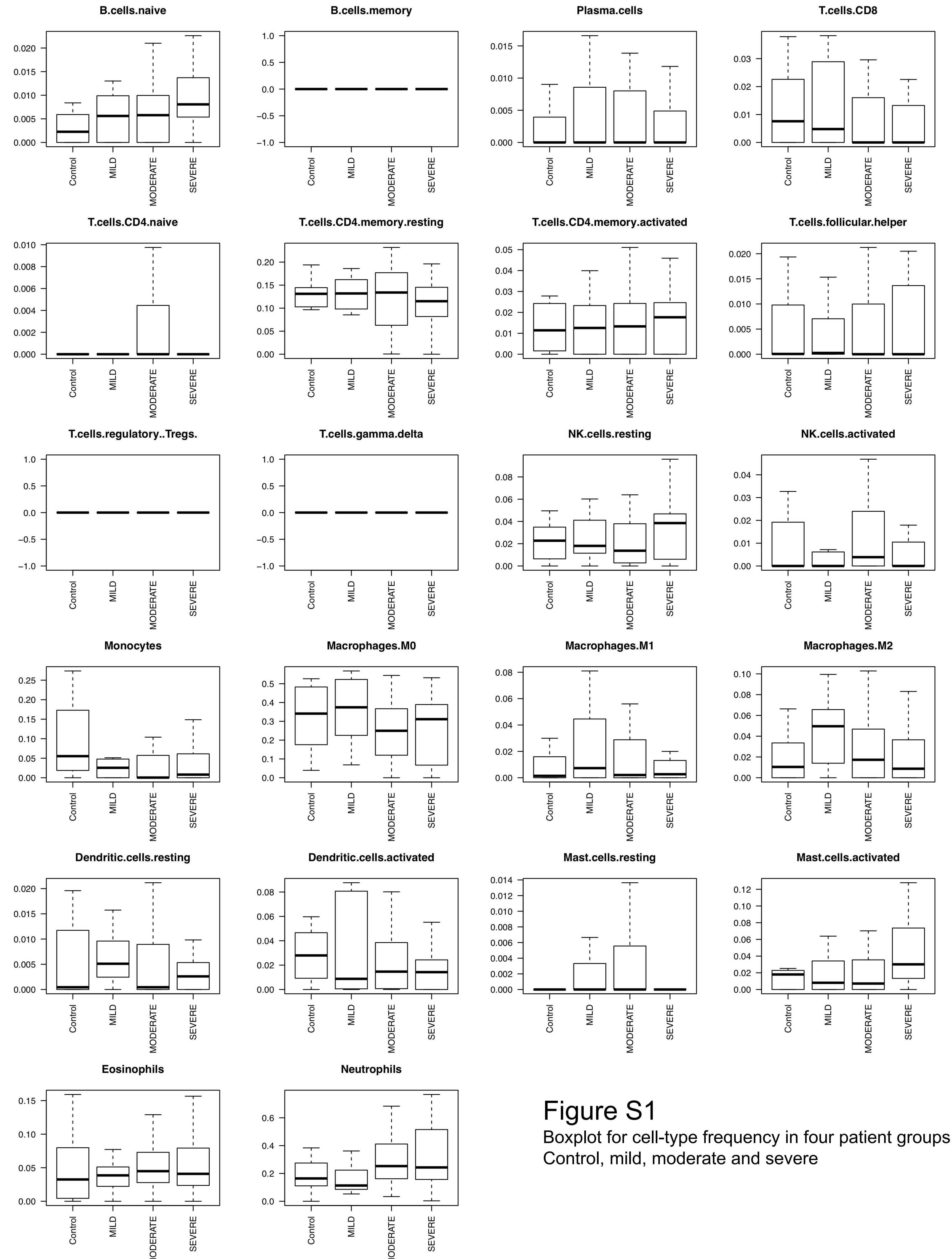

Figure S1

Boxplot for cell-type frequency in four patient groups: Control, mild, moderate and severe

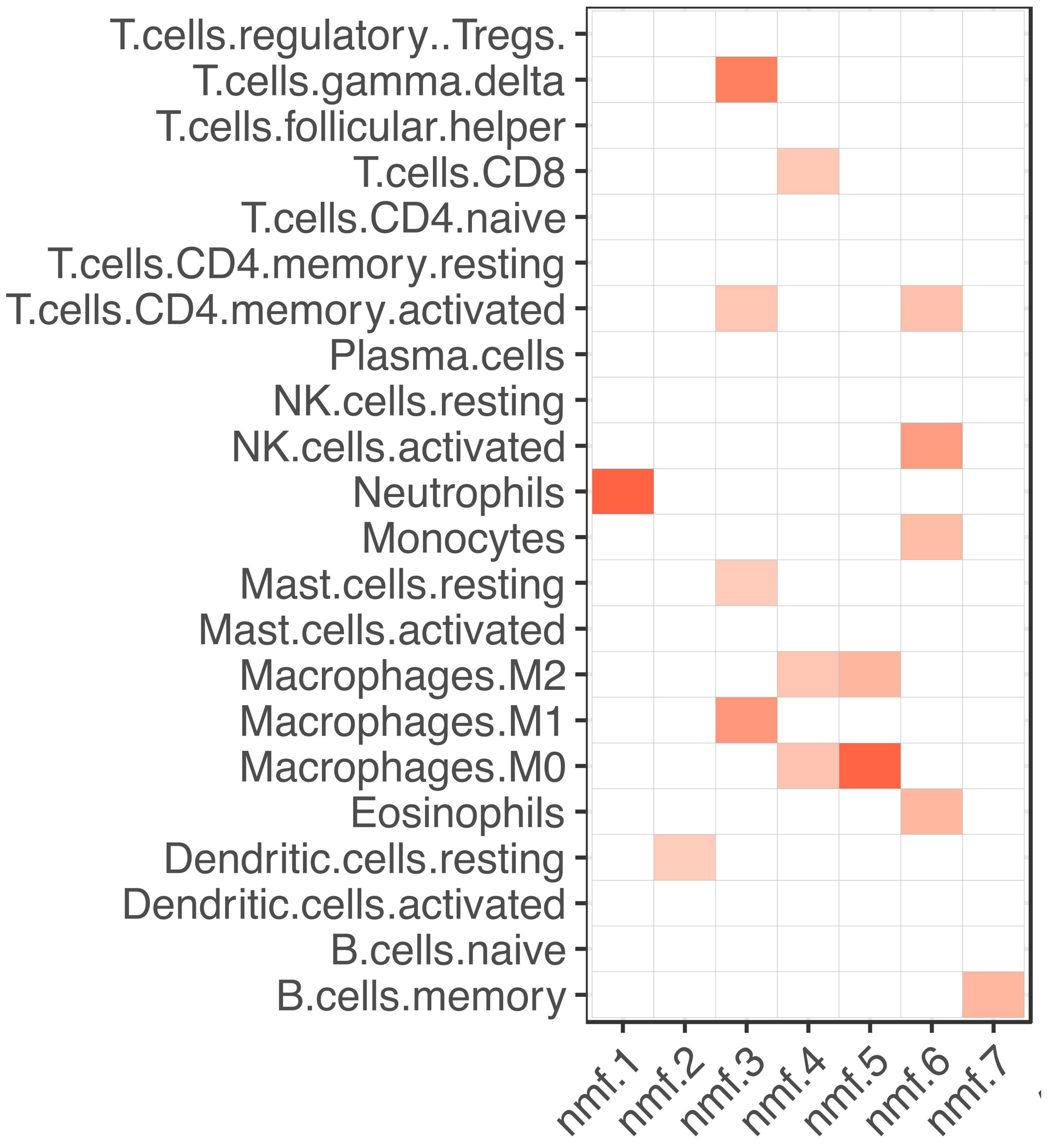

Figure S2:

Correlation between NMF weight matric and cell-type fraction

Figure S3: microbe abundance correlation with clinical information.

A

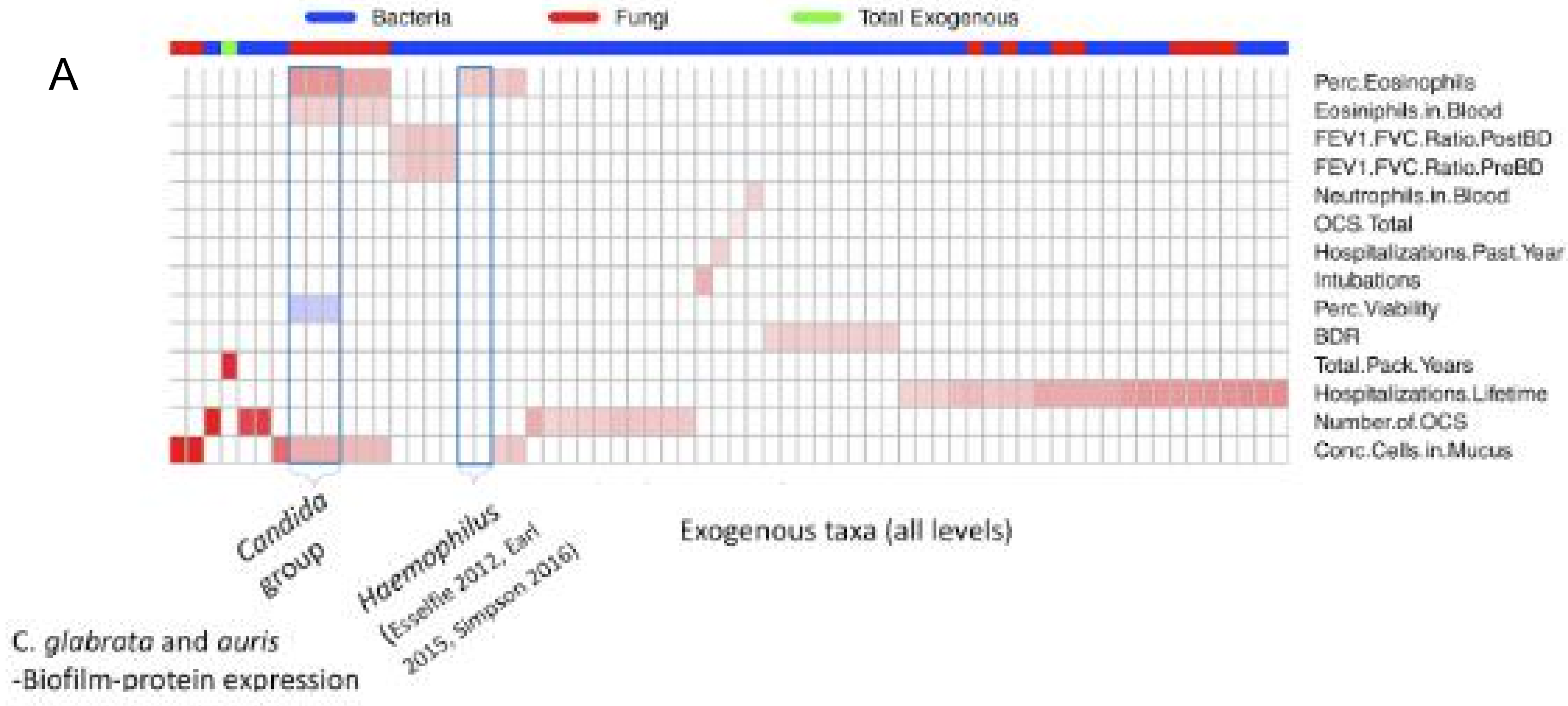

B

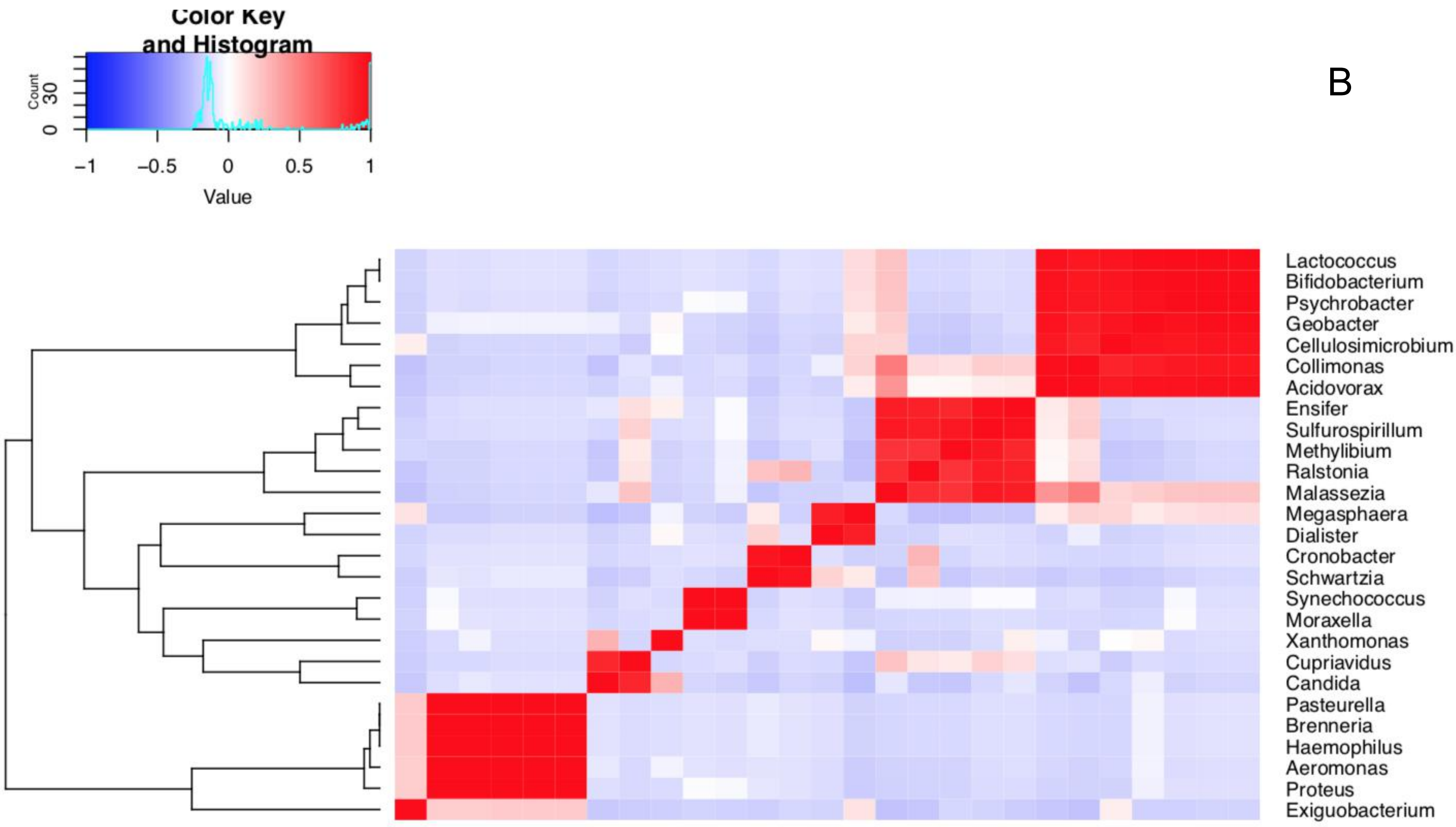

Figure S4: correlation between gene and microbe topic fraction in patients and clinical information.

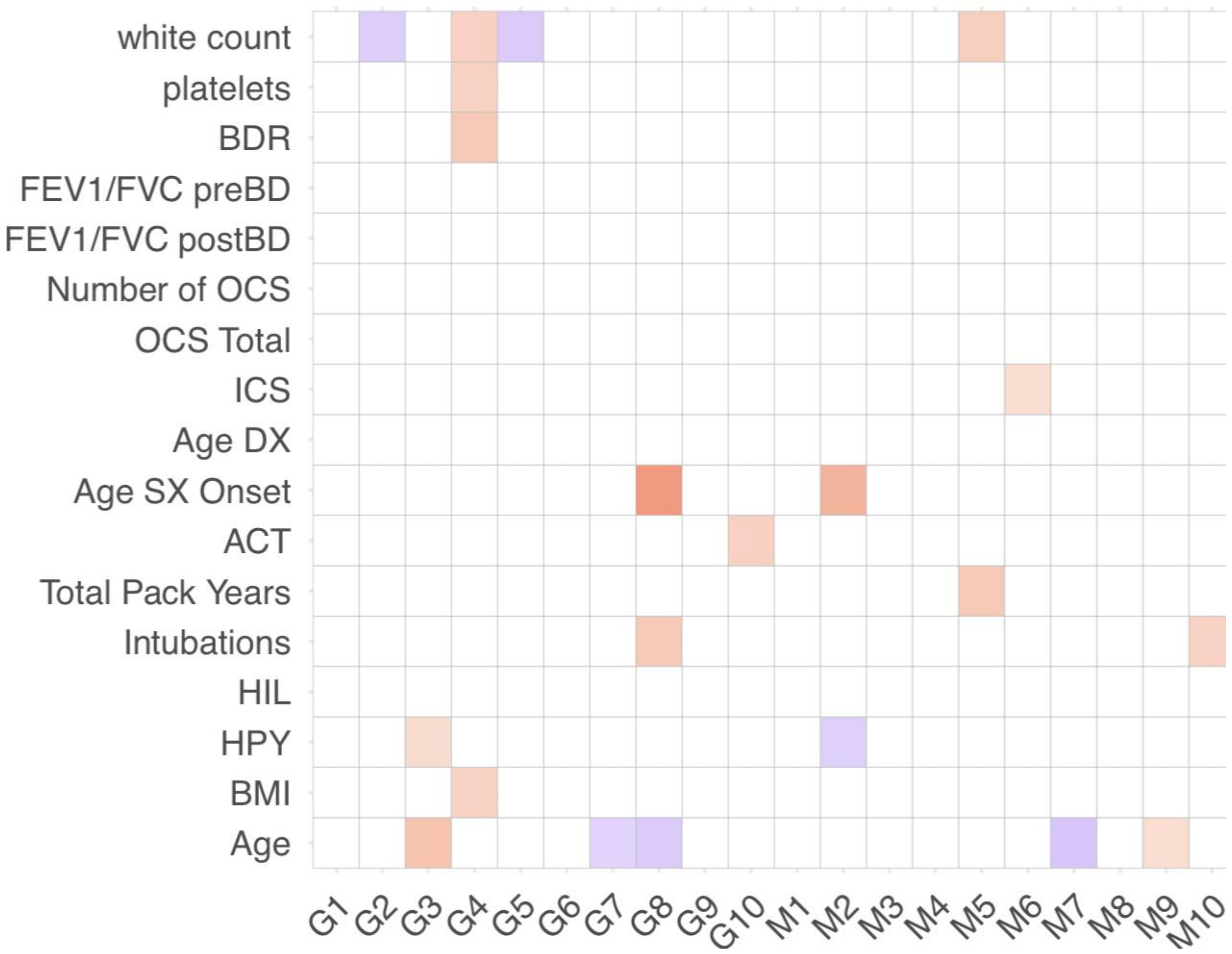

Figure S5: microbe co-abundance network using the correlation with raw microbe abundance (A: in network view; B: heatmap view).

A

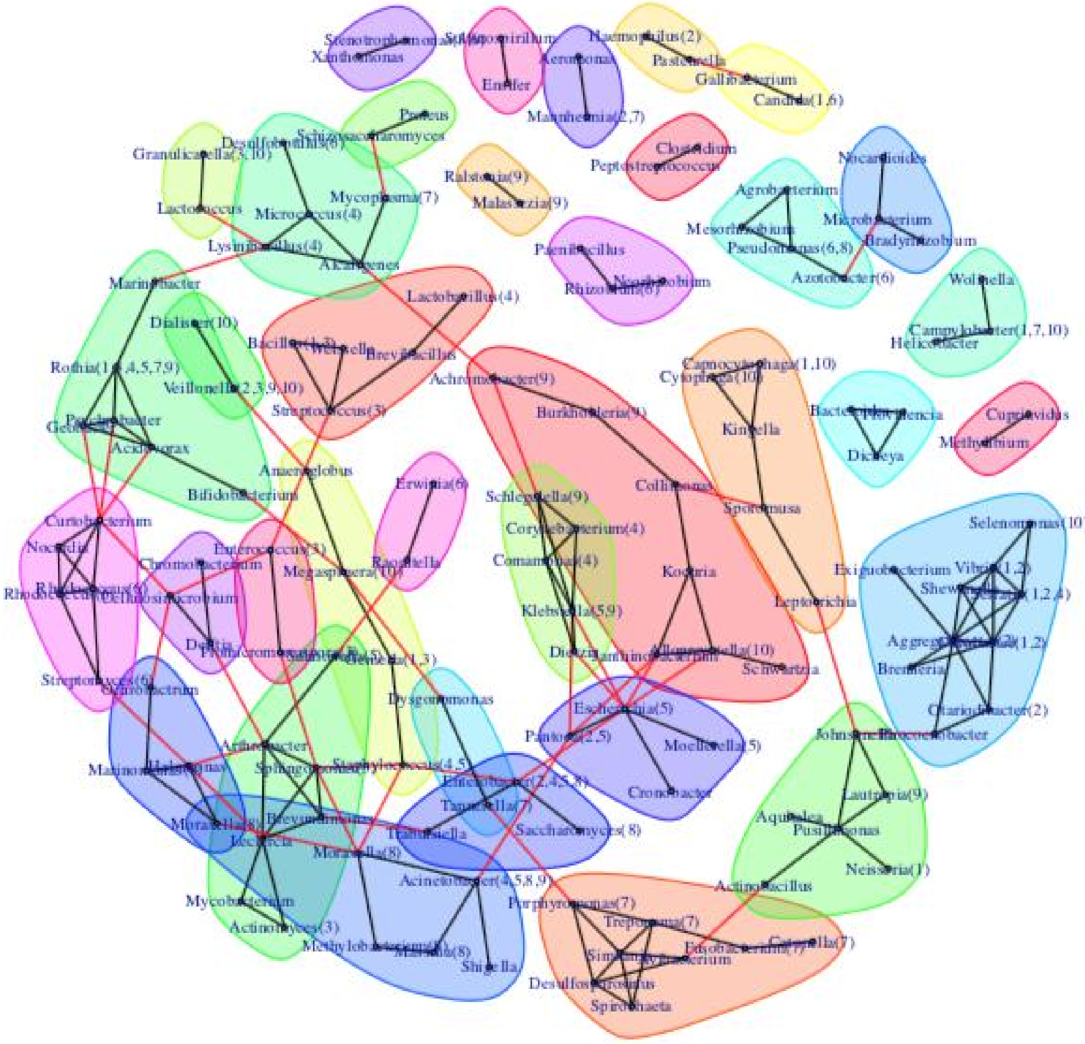

B

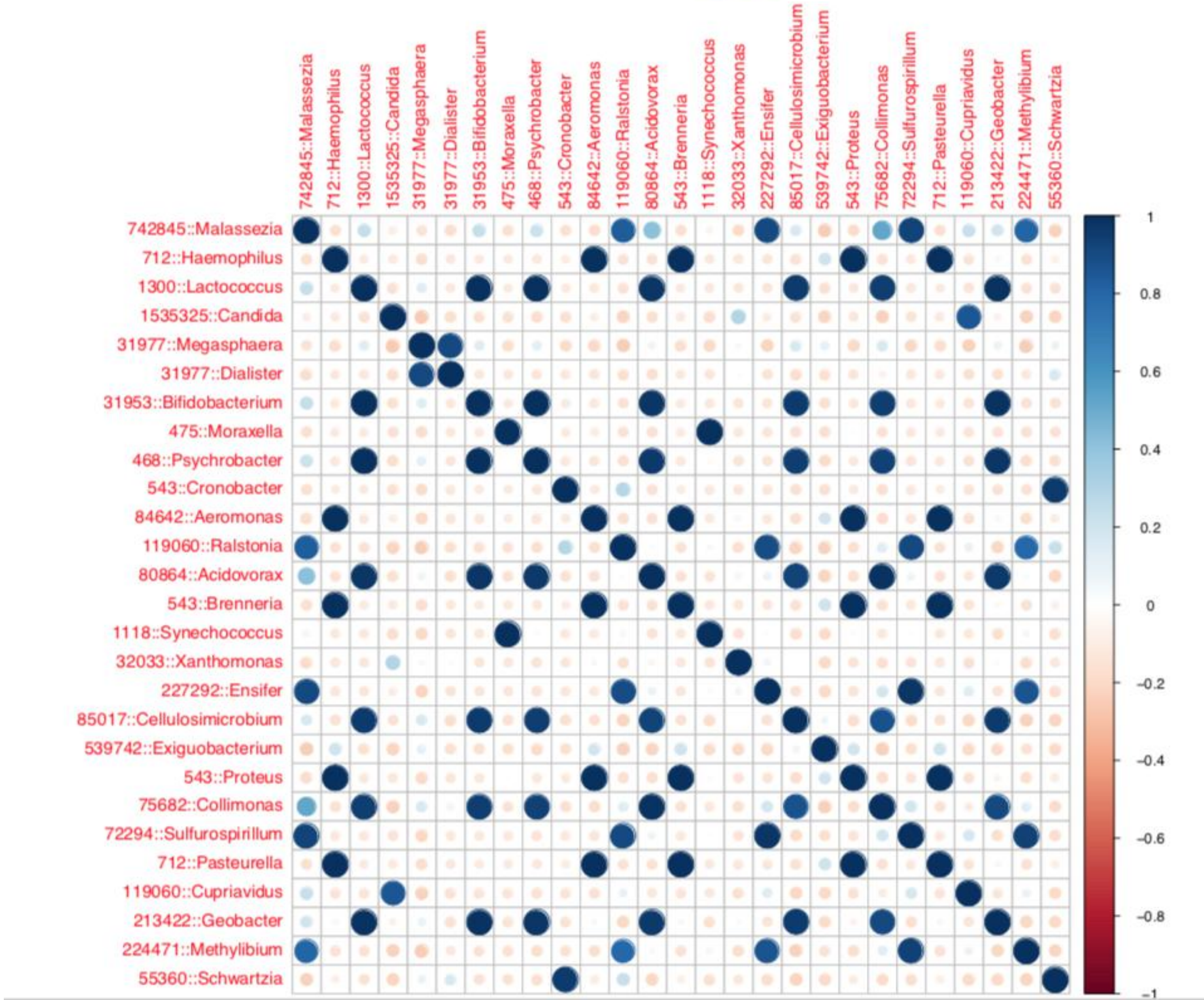

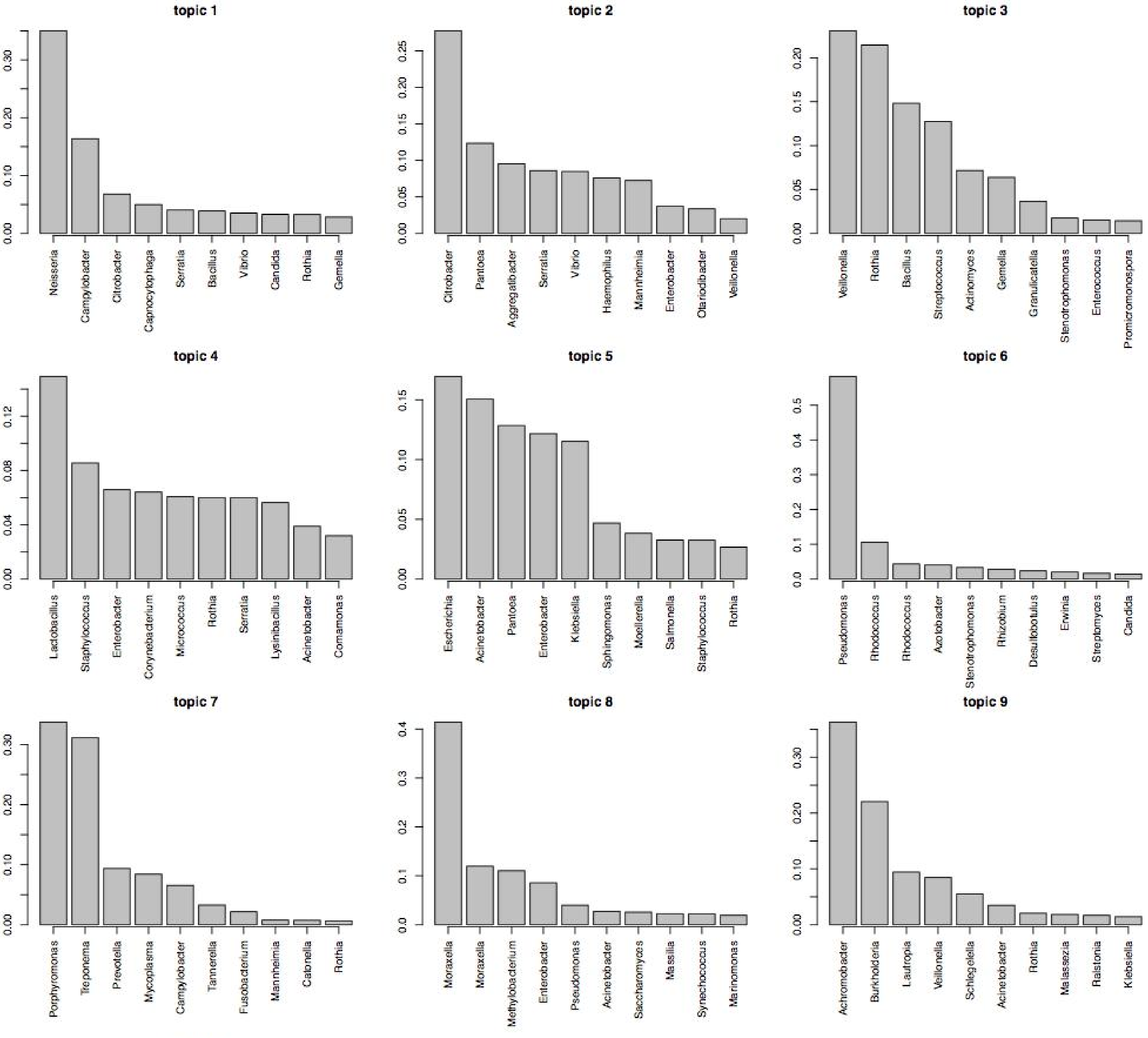

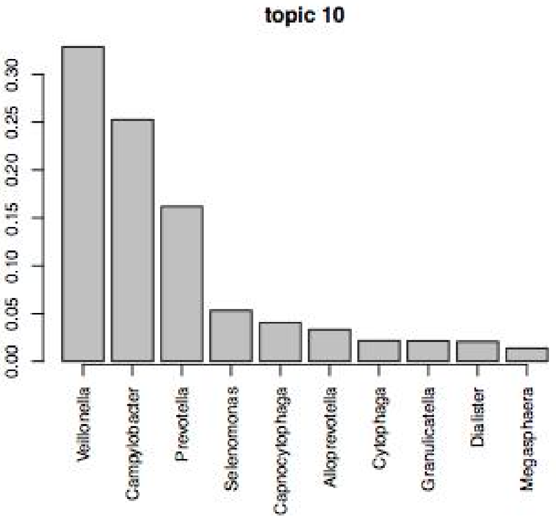

Figure S6
Top weighted microbe in the microbe topics.

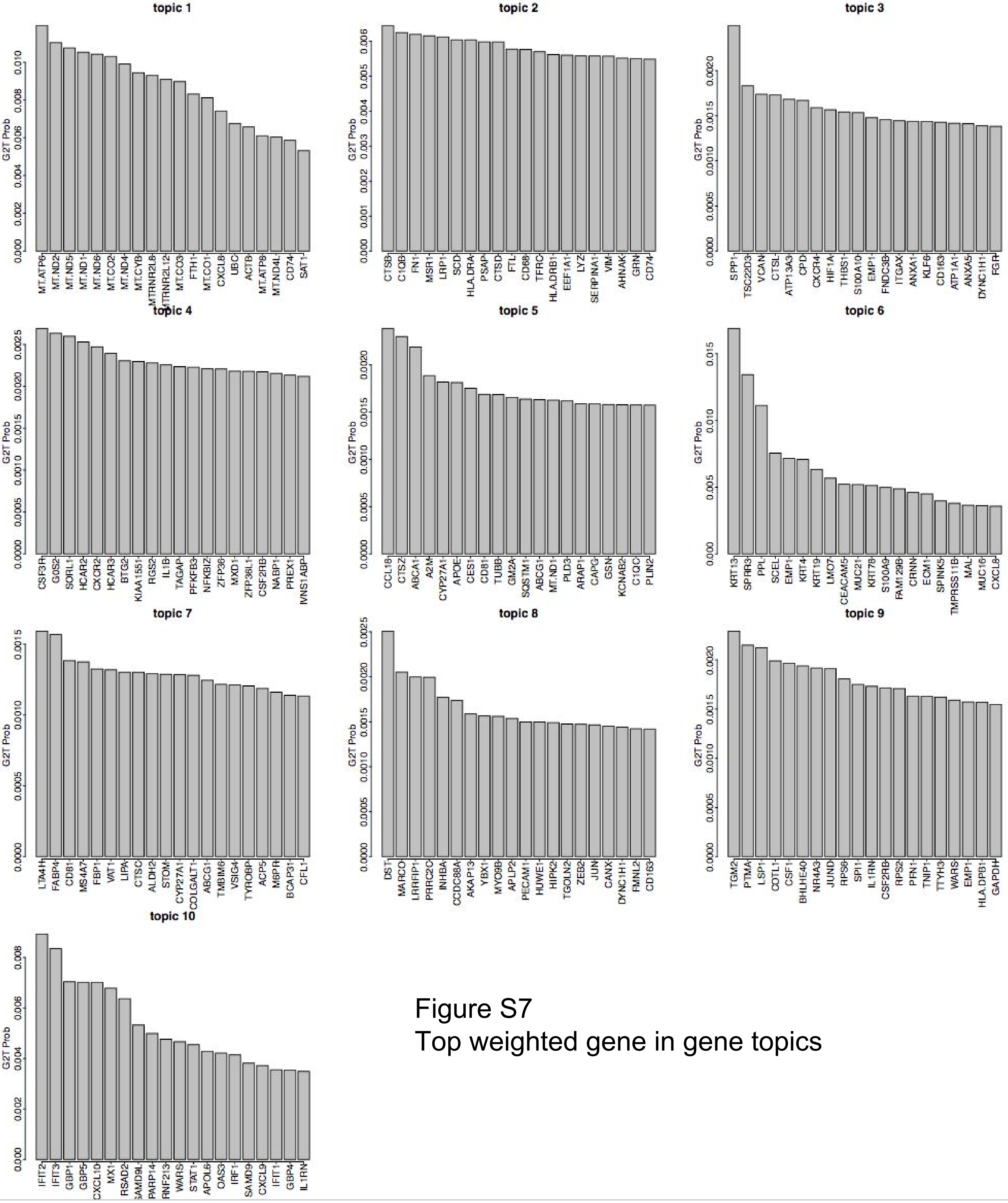

Figure S7
Top weighted gene in gene topics

Figure S8:
Heatmap of Gene topic fraction in patient (A) and
Gene enrichment analysis of top weighted gene in topic 4 (B).

A

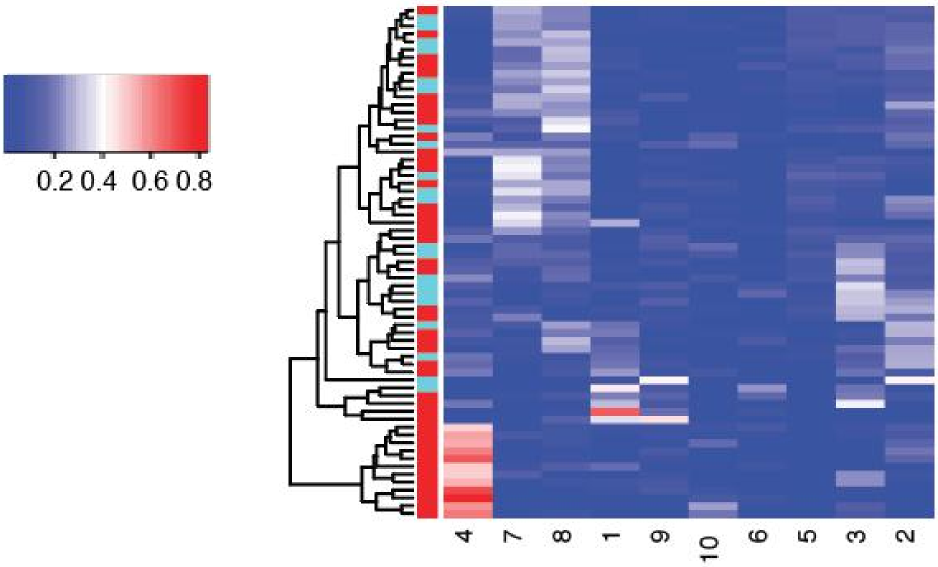

B

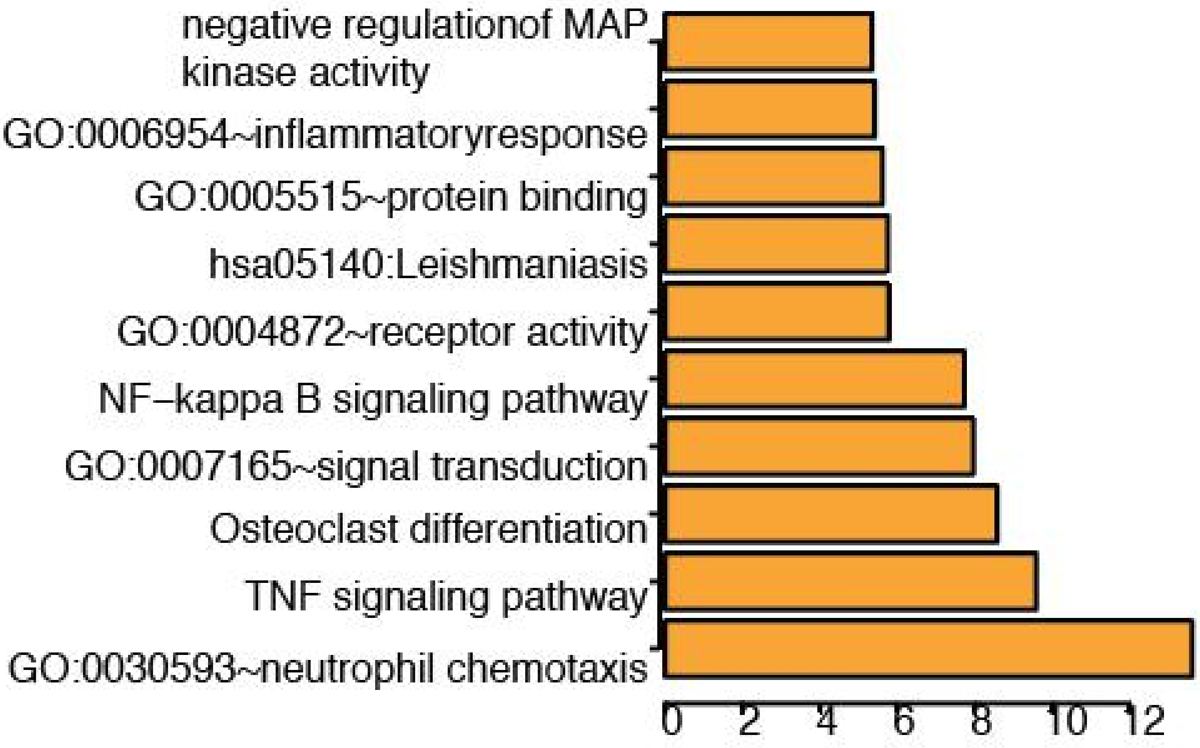

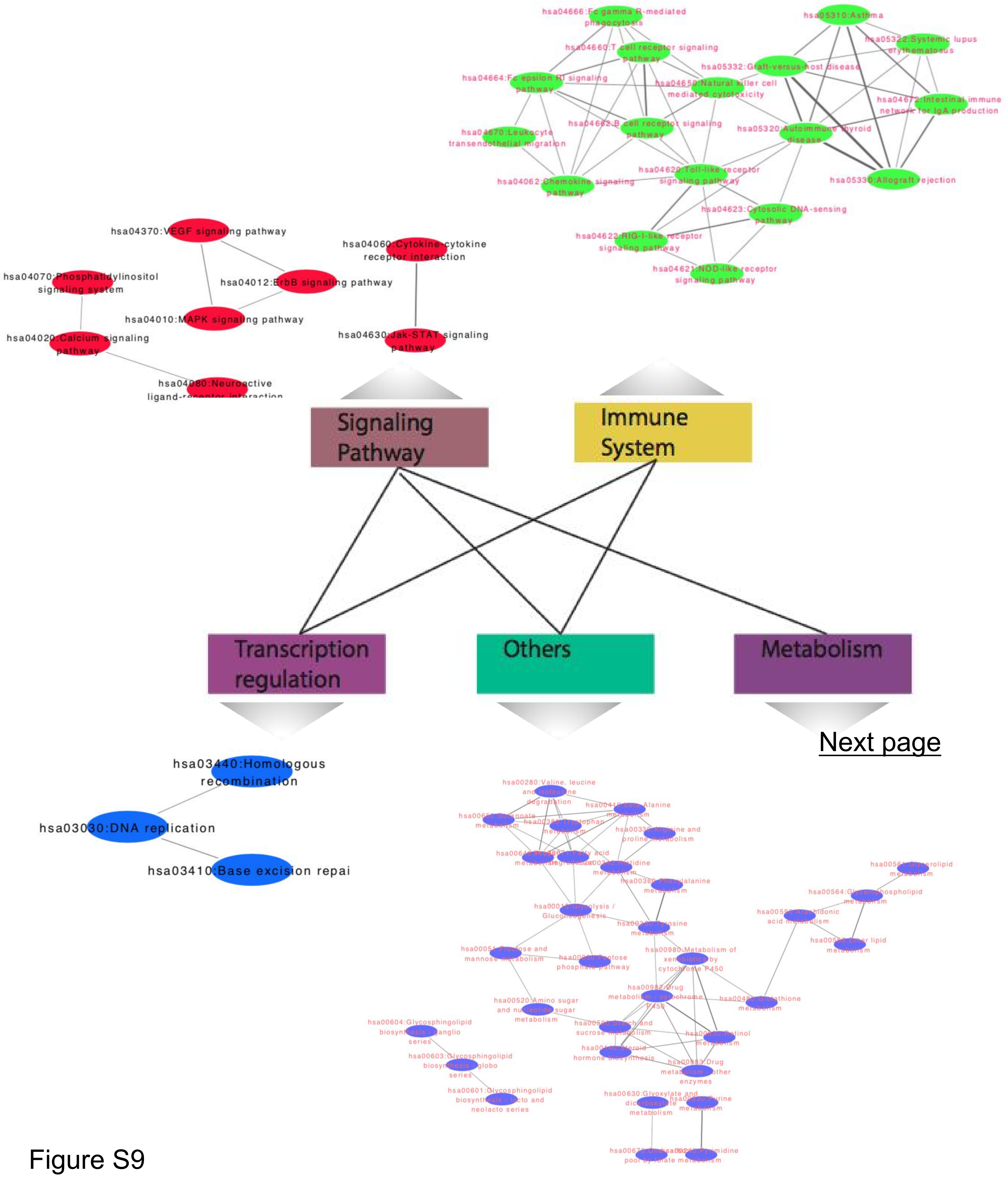

Next page

Figure S9
Function annotation finds the enriched gene pathway

Figure S9 continued: Metabolism network.

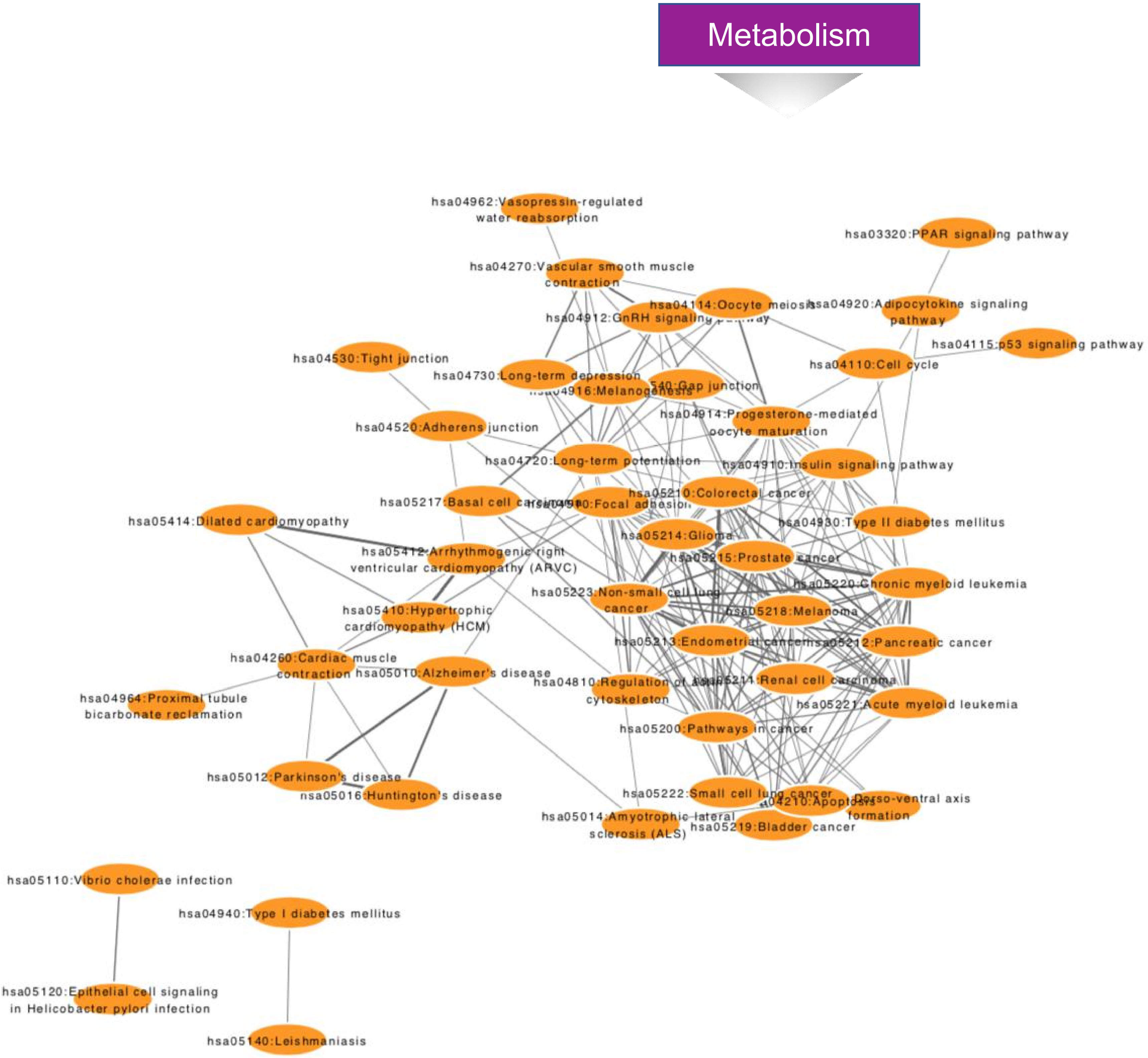

Fig S10 comparison between LDA, NMF and PCA

A

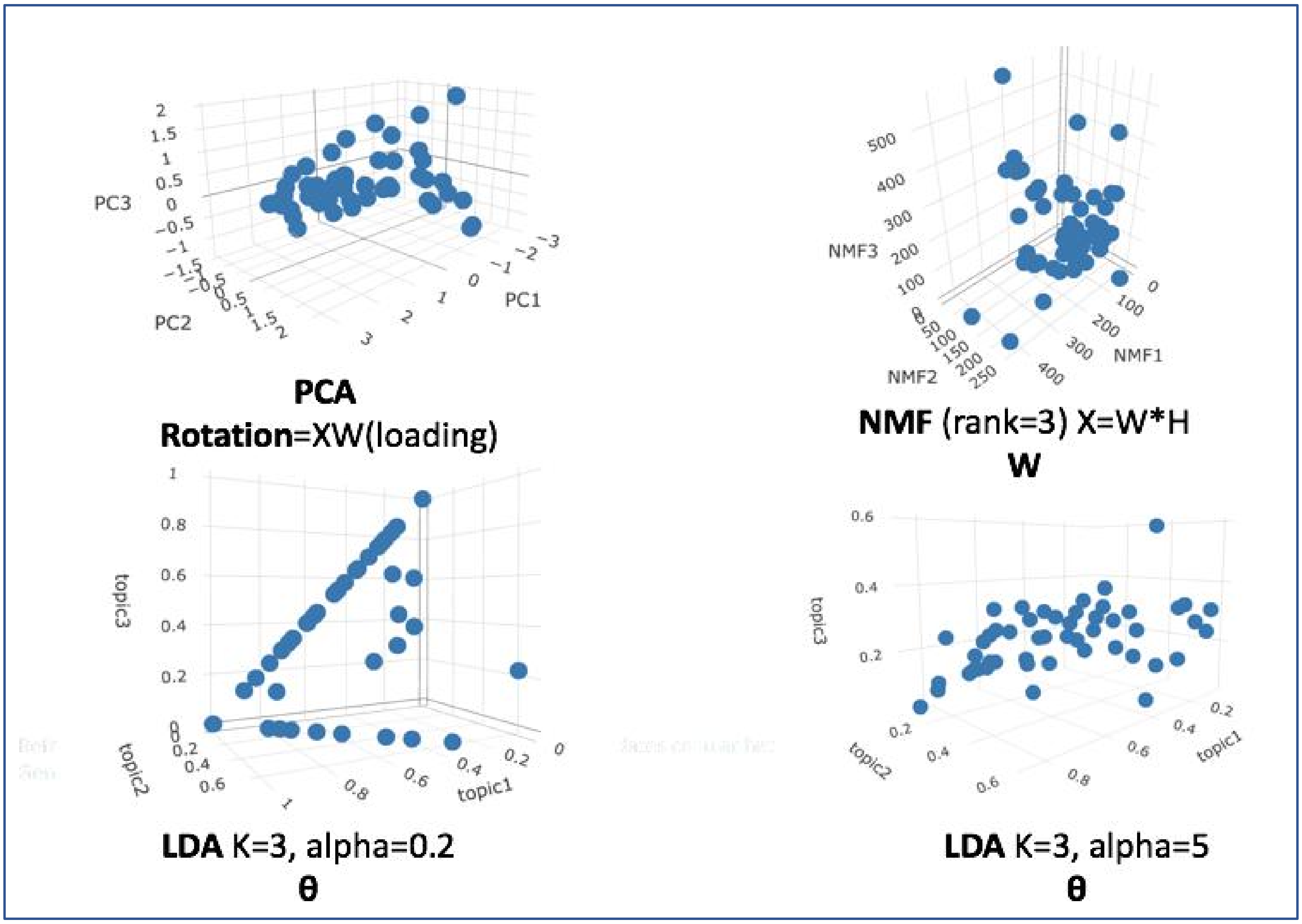

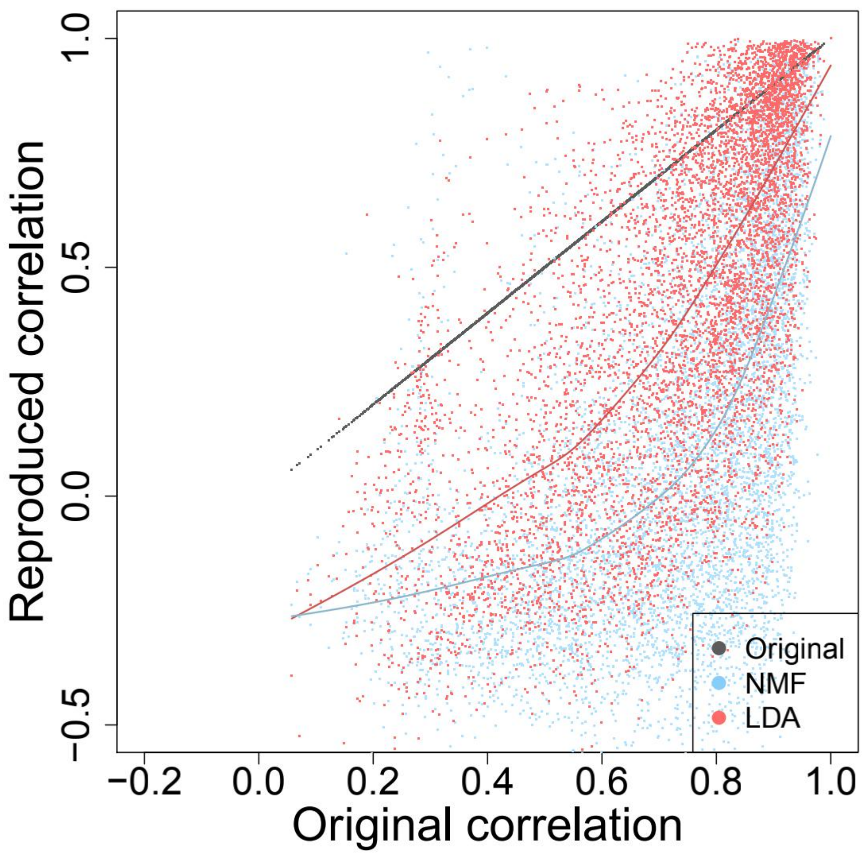

B Human gene

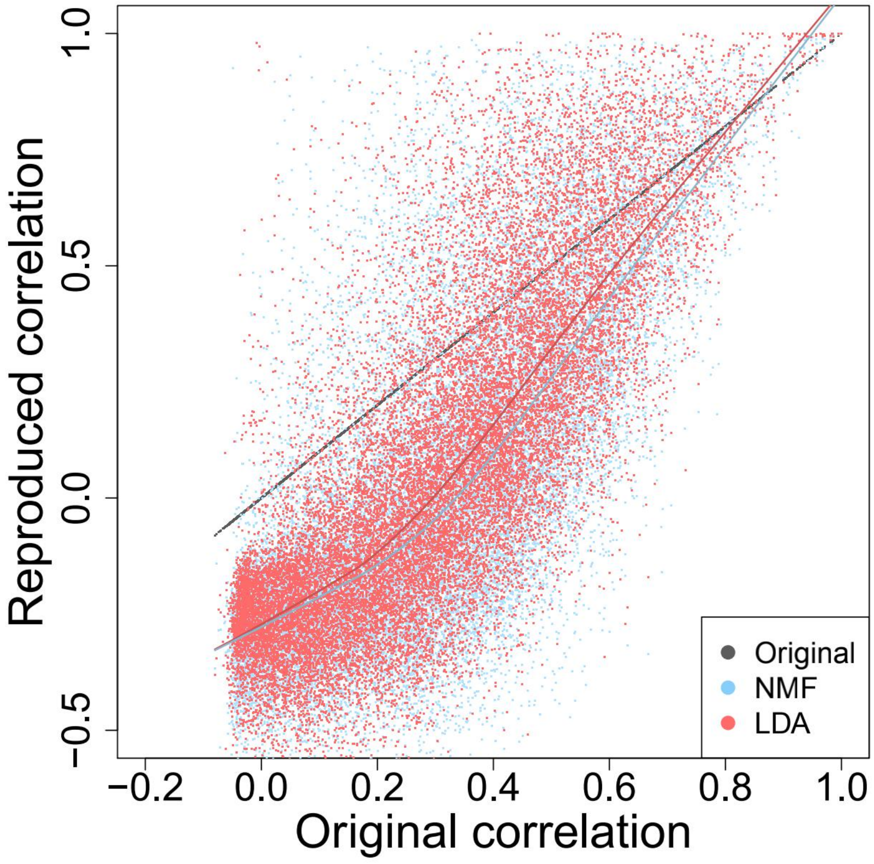

C Microbes

Fig S11 Diagram of cross-validation

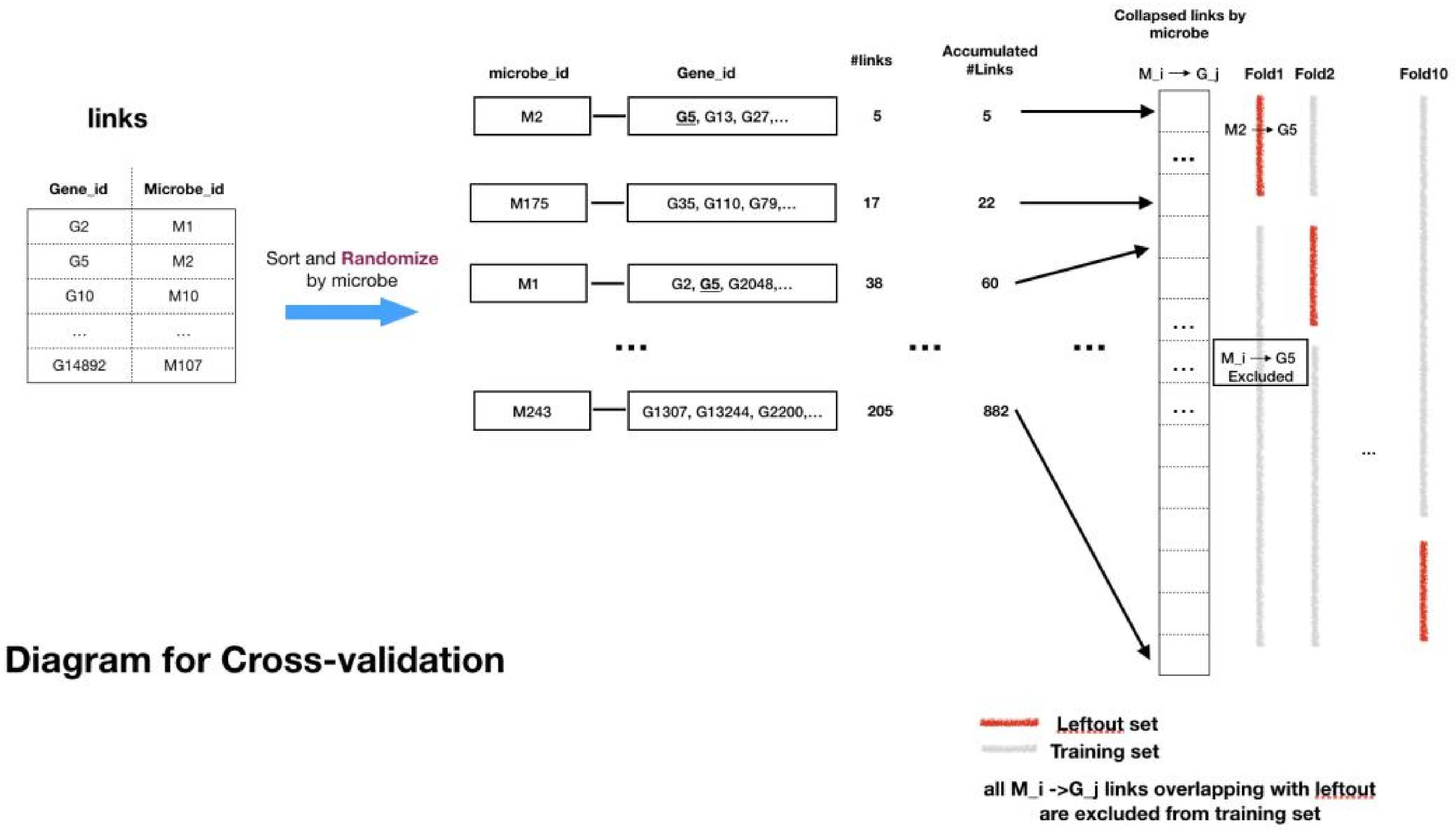

Supplement: Supplementary file 1 — Additional file 1:Figures S1-S11. Supplemental Figures. [file 13059_2020_2033_MOESM1_ESM.pdf]
