# Supplementary material for: Approaches for integrating heterogeneous RNA-seq data reveal cross-talk between microbes and genes in asthmatic patients
Source: Genome Biol. 2020 Jun 22;21:150. doi: 10.1186/s13059-020-02033-z (PMC7310008; doi:10.1186/s13059-020-02033-z)
Supplement: Supplementary file 5 — Additional file 5: Review history. [file 13059_2020_2033_MOESM5_ESM.docx]

Review History

**First round of review**

**Reviewer 1**

**Comments to author:**

In this paper, Spakowicz et al. analyzed gene expression data including both human and microbial genes from sputum samples. They investigated the relationships among clinical data, relative abundance of cell types and microbial species. The study is novel and the results are interesting. The study definitely contributes to our understanding of the interplay among clinical variables, human genes and microbiome. I have a few suggestions to improve the study.

Major concerns:
1.     I understand the decomposition G = F*S on line 28, page 4 in the main text. However, I cannot understand the equations G = F*E on line 52, page 9, and the equation S = G*F on line 4, page 10. I think there are something wrong in the “Methods” section.
2.     There are many places stating statistical significant associations among some quantities of interest, such as lines 7-14, page 5, without stating the methods for adjusting multiple testing. What are the criteria to make these claims?
3.     The authors clearly showed that the estimated cell type fractions using reference gene expression for different cell types are meaningful. However, the decompositions based on LDA do not have clear biological meanings as shown in lines 19-59, page 5. The same should hold for decomposition of microbial fractions. Since most of the unsupervised decompositions do not have clear meanings, I do not understand what we can learn from the correlations between these LDA components and clinical variables.

Minor points.

1.     N_p is not defined although I can guess its meaning from the context. Please define.
2.     Line 40, page 5, please explain how the single-cells are clustered, what distance measure and what clustering algorithms were used.

**Reviewer 2**

**Comments to author:**

Review of Spakowicz et al. Approaches for integrating heterogeneous RNA-seq data reveals cross-talk
between microbes and genes in asthmatic patients

Using RNA-seq data of lung sputum samples collected from 115 asthmatic patients, this manuscript describes the mapping of sequence reads human cell types and the microbiota, then applying Latent Dirichlet Allocation (LDA), a machine learning algorithm, and other approaches to understand the underlying relationships in the data. While the overall goal of integrative analyses of human and microbiota datasets is potentially interesting, the study has many flaws and the manuscript needs to massively re-written before being acceptable for publication. Below are listed major revisions which, in their totality, argue for a rejection of this manuscript in its present form.

Major Comments

1.     A number of papers have used LDA in the clustering of human gene expression data for other diseases such as cancer survival predication,  (https://www.ncbi.nlm.nih.gov/pubmed/31730620). This manuscript does not provide any context or review of the application of LDA or other approaches for data reduction and integration.

2.     If the goal is to demonstrate the value of LDA in noise reduction then there needs to be comparisons to other existing methods.

3.     There is a very poor description of the underlying data-set. No information is provided on the patient cohort, such as the time of sampling during disease events (i.e. stable vs asthmatic events), demographics, etc. Was the study longitudinal or cross-sectional?

4.     Mapping RNA-seq reads to both the microbiome and human can be problematic, if there are not proper positive and negative controls during the sample preparation and RNA-seq analyses. What methods and databases were used to map reads to bacterial, fungal or protist pathogens?

5.     Figure 2 is particularly confusions:

a.     Figure 2B and logic for "The reference profiles split into the groups by lineage; for example, those in the lymphoid progenitor line clustered with cluster 2, and the myeloblast progenitor line co-clustered with cluster 4" is unclear. There is a need to explain the LM22 gene signature and all gene signatures or topics. What are the driver genes in the top pathways?

b.     Figure 2C needs to show P-values and explain outliers in the text.

c.     Figure 2D - In the Results, the number of samples and how these were selected need to be explained.

d.     Figure 2E - What are key elements of the topics? A Supplementary Table with the topic composition as well as some description in the text is necessary.

e.     Figure 2F - Abbreviations should be explicit in the captions. This Figure is supposed to support the statement: "For example, the fraction of T-regulatory cells negatively correlated with the number of hospitalizations per year, suggesting a beneficial role of these cells in the management of asthma." What evidence or analysis suggests the T-reg faction predicts hospitalizations? Does an AUC curve show the relative weighting and strength of the topics in predicting particular clinical variables?

6.     Page 7 Line 57 - Mentions "Supplementary Table X" which is not in the submission.

7.     Page 8 Line 43 - Mentions "Supplementary Figure Z" which is also not in the submission.

8.     Page 8. Line 7 - What is the statistical significance of the correlations? An important caveat for the whole manuscript is that correlation does not imply causation. How would the authors translate their correlations into useful insights into disease biomarkers, diagnostics or therapeutics?

9.     Page 8 and throughout. There is an ad hoc mixture of Results and Discussion sections in the manuscript. The manuscript needs to be restructure with a factual Results section, then relating those findings to the existing literature in the Discussion.

10.     Page 8 Line 35 - What is the clinical measurement of "asthma severity"? It is likely a multi-factorial index of patient wellness and lung function.

11.     Page 8 Line 37 - The observation that lung Proteobacteria is associated with asthma or only lung diseases, such as COPD, is not novel (see https://www.ncbi.nlm.nih.gov/pubmed/31612122).

12.     Page 8 Line 53 - The statement "How much cross-talk exists between microbes and human cells in the airway remains contentious [17]" is disingenuous. There are many papers linking bacterial metabolites to modulation of human immunity (for a recent review, see: https://www.ncbi.nlm.nih.gov/pubmed/31501577 ).

13.     Page 8 Line 53-61 & Page 9 Line 1-29. The LDA-Link section is highly flawed because its training set is a subset of the existing study data built on the same LDA associations. A true training set should be from a different data-set. Also, there is no mentioned of cross-validation of the model.

14.     Page 9 line 33-50 - This review of previous work does not clearly different between human receptor cross-talk with the microbiota versus innate immunity or auto-immunity response to pathogens. This should be in the Discussion as well. However, there is still a question about the novelty of the LDA approaches given that the uncovered relationships are known.

15.     Page 10 Line 29-35. The statement, "A limited number of reports have investigated the changes in the lung microbiota between healthy, non-smoking and smoking individuals as well as in patients suffering from cystic fibrosis, chronic obstructive pulmonary disease, or 317 asthma [2, 28-30]. Despite emerging data on the airway microbiota, little is known about the role of the lung microbiome in modulating pulmonary mucosal immune responses…" is inaccurate. A search of PubMed using the term "lung microbiome asthma" retrieved 260 citations. For the "lung microbiome" alone, there are 1480 citations.

**Authors Response**

**Point-by-point responses to the reviewers’ comments:**

Comment

*Response*

Reviewer #1: In this paper, Spakowicz et al. analyzed gene expression data including both human and microbial genes from sputum samples. They investigated the relationships among clinical data, relative abundance of cell types and microbial species. The study is novel and
the results are interesting. The study definitely contributes to our understanding of the interplay among clinical variables, human genes and microbiome. I have a few suggestions to improve the study.

Major comment 1. Accuracy of equations
Reviewer comment
I understand the decomposition G = F*S on line 28, page 4 in the main text. However, I cannot understand the equations G = F*E on line 52, page 9, and the equation S = G*F on line 4, page 10. I think there are something wrong in the “Methods” section.
*Author response
Thank you for the comment. We carefully reviewed these equations for accuracy and have adjusted the notation in an effort to improve clarity.
Excerpt From
Methods, Supervised deconvolution, pg 11 The gene table (G) was deconvolved using the transcriptomes from 22 flow cytometry-sorted and sequenced immune cell types using the CIBERSORT tool [7]. Briefly, characteristic gene expression patterns for each cell type were used to estimate the fraction of each cell type in the sample by solving for the equation:
G = F S’
Where G is the Gene table of human protein-coding gene expression from the exceRpt pipeline, F is the Cell Fraction table, and S’ is the reference cell-type gene expression signature from experimentally-isolated and sequenced cell types. Support Vector Regression was used to perform variable selection, reducing the number of characteristic genes used to distinguish cell types and thereby reducing overfitting. The above equation was then solved to provide an estimate of F. P-values for the fit of F to G demonstrated that all samples were significant at α = 0.05. Following the solution of F, the cell type expression, S, expanded to all the other, non-signature genes, were inferred using the same equation. [5]*

Major comment 2. Reporting the method of false-discovery rate correction
Reviewer comment There are many places stating statistical significant associations among some quantities of interest, such as lines 7-14, page 5, without stating the methods for adjusting testing. What are the criteria to make these claims?
*Author response Correlation p-values were corrected for multiple-hypothesis testing following the method of Benjamini and Hochberg. We now explicitly define the p-values for each claim, mention the FDR correction method with the first correlation, and also include the correction method in the Methods.
Excerpt From
Methods, page 13,
Results, page 5 Correlation p-values were corrected by the method of Benjamini and Hochberg [54].
e.g.
For example, the fraction of T-regulatory cells negatively correlated with the number of hospitalizations per year (p-value = 0.002, all correlations FDR corrected, see Methods), suggesting a beneficial role of these cells in the management of asthma.*

Major comment 3. Biological meaning of LDA topics
Reviewer comment Major comment 3. The authors clearly showed that the estimated cell type fractions using reference gene expression for different cell types are meaningful. However, the decompositions based on LDA do not have clear biological meanings as shown in lines 19-59, page 5. The same should
hold for decomposition of microbial fractions. Since most of the unsupervised decompositions do not have clear meanings, I do not understand what we can learn from the correlations between these LDA components and clinical variables.
*Author response We agree that the deconvolution to immune cell fractions is a more interpretable unit than an LDA topic, and that this feature is important when applied in the context of microbes. However, we note the relevance of LDA topics in Figure 2D, where a subset of severe asthmatics associated with LDA topic 4, rather than a cell fraction. Despite this being less interpretable than a cell fraction, it may be highly relevant. A more thorough analysis of genes in this topic may indicate a phenotype that is a function of multiple immune cell types, the presence of some cell type not represented here, or some other phenomenon. A similar logic should hold for the microbe decomposition.

We argue that there is no parallel method to reduce the dimensionality of microbes as for the gene deconvolution to cell types. Therefore, we applied LDA both for consistency with the gene table analysis, but also because LDA performs well with data of these types. A more thorough evaluation of its performance is included in this version of the manuscript. A strong microbe-topic to clinical variable correlation could indicate some structure within the sputum microbes, perhaps an ecological niche or collection of organisms that trigger similar innate immune response pathways.

We have adjusted the text when regarding microbial decomposition methods to better define how we conceptualize the relationship with other variables.
Except from Results, Dimensionality reduction for microbes: clustering and networks, page 6 Similarly, we could denoise the microbe table using a co-abundance network, by correlating the genus-level abundances (R(𝑴∙, 𝒎, 𝑴∙, 𝒎) and identifying significant modules (Figure S5). An interpretation of these modules is that they define metabolic niches, where microbes either directly compete for metabolites or there is interdependency in metabolite production. Such networks could be created from other tables, such as the topic distribution of microbes (R(𝝋𝑴∙, 𝒎, 𝝋𝑴∙, 𝒎) for all the topics) (Figure 3D). These modules represent another unit that could be related to the clinical information (C) and the cell-type fractions (F).*

Minor comment 1. Definition of Np
Reviewer comment Minor comment 1. N_p is not defined although I can guess its meaning from the context. Please define.
*Author response N_p is now defined in the Methods section, “Data tables notation.”
Excerpt from Methods, page10: N_p is the number of documents (patients ), N_w is the number of words in a document, N_k is the number of topics (set as 10), N_v is the total number of words in a dictionary; p,w,k,and v denote an index of documents, an index of word in a document , an index of topics and an index of word in a dictionary respectively;*

Minor comment 2. scRNAseq methods
Reviewer comment Minor comment 2. Line 40, page 5, please explain how the single-cells are clustered, what distance measure and what clustering algorithms were used.
*Author response We added text to more clearly describe the single cell clustering methods.
Excerpt from Methods, page 11: “Data were cleaned, processed, aligned, and quantified following default parameters of the SINCERA pipeline [34]. Briefly, the data are processed to filter genes with poor expression across all cells and cells with poor expression across most genes (>= 5 FPKM in 2 cells of the sample, >=1000 genes in a cell) and then transformed to z-scores. Clusters were created via principle component analysis and then t-sne of the principle component loadings. The optimal number of clusters was identified by calculating the gap statistic (method = “Tibs2001SEmax”) using the cluster package in R.”*

Reviewer #2: Using RNA-seq data of lung sputum samples collected from 115 asthmatic patients, this manuscript describes the mapping of sequence reads human cell types and the microbiota, then applying Latent Dirichlet Allocation (LDA), a machine learning algorithm, and other approaches to understand the underlying relationships in the data. While the overall goal of integrative analyses of human and microbiota datasets is potentially interesting, the study has many flaws and the manuscript needs to massively re-written before being acceptable for publication. Below are listed major revisions which, in their totality, argue for a rejection of this manuscript in its present form.

Major comment 1. Introduction of LDA
Reviewer comment A number of papers have used LDA in the clustering of human gene expression data for other diseases such as cancer survival prediction, (https://www.ncbi.nlm.nih.gov/pubmed/31730620). This manuscript does not provide any context or review of the application of LDA or other approaches for data reduction and integration.
*Author response We appreciate this comment and have added text to the introduction to add context to LDA.
Excerpt from Introduction, Unsupervised decomposition including LDA, page 3 A number of algorithms have been developed to infer higher-order structure within high-dimensional datasets. Latent Dirichlet Allocation (LDA) was first used to in text mining applications to learn the set of topics in documents and has since been used in a broad range of applications including marketing, genomics, drug-pathway relationships and others[8-11]. Unlike some other unsupervised decomposition methods (e.g. Principle component analysis (PCA), non-negative matrix factorization (NMF)), the result is not a linear decomposition. The topic distributions from LDA are governed by a series of hyper-parameters that define distributions from which the “words” (in this case, genes or microbes) are drawn. This non-linear character has advantages in complex, sparse, or noisy datasets, RNA-seqsuch as sputum RNA-seq or single cell RNA-seq.*

Major comment 2. Comparison of LDA to similar methods
Reviewer comment If the goal is to demonstrate the value of LDA in noise reduction then there needs to be comparisons to other existing methods.
*Author response We agree with the reviewer and have included a thorough comparison to other noise reduction methods, including a new supplemental figure, described below. In brief, LDA provides a sparser decomposition which can achieve more robust and interpretable result compared to PCA and NMF. LDA also performed better for another important aspect of the decomposition, which method best preserves distances within the original data.

Compared with PCA and NMF, the most advantageous feature of LDA is the control of sparsity of low dimensional representations, which gives a robust representation for the noise reduction. LDA can control the sparsity of the document to topic distribution (θ, which corresponding to the feature matrix W in NMF, and rotation value of input X in the PCA) by controlling the hyper-parameters alpha. We demonstrate this using a common machine learning dataset to visualize the effect of sparseness of low dimensional representations of these three methods. For a better and easier visualization, we only decompose the matrix into three-dimensional space. As shown in the figure, by controlling the hyper-parameter alpha, LDA can achieve better sparseness of low dimensional patterns.

Next we use the real data we used in our manuscript to compare another important aspect of the decomposition, which each method preserves distances within the original data. Here we restrict the analysis to NMF and LDA to avoid negative values that are nonsensical in count data. In this comparison, we use the same datasets of bulk-rna seq and exogenous data. For the raw dataset, we calculated the pairwise correlation between the genes or exogenous organisms across patients (“Original"). We then applied LDA and NMF and with the number of topics (10) equal to the NMF rank. We calculate the reproduced correlation between the patients using the document-to-topic distribution from LDA and feature matrix(W) from NMF respectively. If the reproduced correlation is more similar to the original pair-wise correlation, it indicates the low-dimensional representation retains more information after the dimension and noise reduction. From the comparison, we can see LDA retained more information than NMF.

We have updated the text accordingly. Please refer the excerpt from the manuscript below.

Excerpt from Methods, Justification for unsupervised decomposition using LDA, page 10-11 We compared LDA to several commonly-used dimensionality reduction techniques. As a canonical method, principal component analysis (PCA) uses an orthogonal transformation to convert a data matrix into a set of linearly uncorrelated principal components, but it may give negative numbers. Similar to PCA, non-negative matrix factorization (NMF) can be represented by a linear combination of all the variables, but it requires all the matrices have only non-negative elements.
LDA has two advantages over these methods that we demonstrate using a classic machine learning dataset. First, LDA provides a sparser decomposition that yields more robust and interpretable representations. By controlling the hyper-parameter α, LDA achieves better sparseness of low-dimensional patterns (Figure S10). Second, another important aspect of the decomposition is that it should preserve the distances (correlations) between two variables in the original data [52]. The correlations from LDA document-to-topic distributions more closely resemble the linear correlations than the feature matrix(W) from NMF (Figure S11).*

Major comment 3. Description of the dataset
Reviewer comment There is a very poor description of the underlying data-set. No information is provided on the patient cohort, such as the time of sampling during disease events (i.e. stable vs asthmatic events), demographics, etc. Was the study longitudinal or cross-sectional?
*Author response We have included additional details in this version, including a supplemental table that describes the cohort.
Excerpt from Methods, Patient cohort, page 10. The patient population in this study received treatment at the Yale Center for Asthma and Airway Diseases before 2016. Included were patients classified as having mild, moderate or severe asthma, as defined by the amount of fluticasone or equivalent per day to control symptoms (mild = > 200μg, moderate = 200 − 800μg, severe = > 800μg). Most patients were young (median age 46) female (~75%) and white (60%). A single sample was used from each patient, not within two weeks of an exacerbation event. Control samples from 13 healthy individuals were included (Supplemental Table S2).*

Major comment 4. Alignment to human and exogenous genomes
Reviewer comment Mapping RNA-seq reads to both the microbiome and human can be problematic, if there are not proper positive and negative controls during the sample preparation and RNA-seq analyses. What methods and databases were used to map reads to bacterial, fungal or protist pathogens?

*Author response We agree that this is computationally challenging, and our concern for spurious alignments and false discovery motivated our use of the tool exceRpt (https://github.com/gersteinlab/exceRpt). Unlike methods that align both to human and exogenous databases simultaneously, exceRpt proceeds serially, filtering reads through databases that reflect the most likely sources of reads. In particular, first reads are aligned to contaminant databases, then human ribosomal RNA, then the human genome, etc. Only after filtering these reads are the unaligned mapped to exogenous databases, starting with the well-curated Ribosomal Database Project rRNA genes, and then finally to exogenous genomes. Multimapping reads are retained at the last-common-ancestor nodes as is done by other methods, e.g. Kraken \cite{kraken}.
Excerpt from Methods, RNAseq processing by exceRpt, page 10. Clipped, collapsed reads were filtered through the Univec database of common laboratory contaminants and a human ribosomal database before being mapped to the human reference genome (hg19) and pre-miRNA sequences using STAR [31]. Reads that did not align were mapped against a ribosomal reference library of bacteria, fungi, and archaea, compiled by the Ribosome Database Project [32], and then to genomes of bacteria, fungi, plants, and viruses, retrieved from GenBank [32]. In cases where RNAseq reads aligned equally well to more than one microbe, a “last common ancestor” approach was used, and the read was assigned to the next node up the phylogenetic tree, as performed by similar algorithms [7, 33].*

Major comment 5a. Explanation of Figure 2B
Reviewer comment Figure 2 is particularly confusions:
a. Figure 2B and logic for "The reference profiles split into the groups by lineage; for example, those in the lymphoid progenitor line clustered with cluster 2, and the myeloblast progenitor line co-clustered with cluster 4" is unclear.
*Author response The text describing this result has been heavily edited for clarity.
Excerpt from Results, Evaluation of deconvolution results by scRNAseq, page 5 In order to identify the clusters, we co-clustered the reference cell types with the scRNA-seq data (Figure 2B, third panel); the reference cells grouped by lineage each with a different scRNA-cluster. For example, the references cells of lymphoid origin (e.g. T-cells, NK cells) clustered together with one of the scRNA-seq clusters, and the reference cells of myeloblast origin (e.g. neutrophils, eosinophils, macrophages) clustered together and with another scRNA-seq cluster.*

Major comment 5b. Description of the LM22 dataset
Reviewer comment There is a need to explain the LM22 gene signature and all gene signatures or topics. What are the driver genes in the top pathways?
*Author response The lm22 dataset and a description of its signature genes has been published and described thoroughly elsewhere. We more carefully refer to this work in the manuscript to avoid confusion.
Excerpt from Results, Analysis of human-aligned reads, page 4. ...we deconvolved the Gene table (G) into a table of fractions of component cells type (F) and cognate cell-type signatures (S) by solving the formula G = F S. This method relies on knowing the signature gene-set in each cell type, for which we used high-quality reference sets from experimentally-isolated and sequenced circulating immune cells [7]. To validate that these circulating immune cells behaved similarly to those in sputum, we generated additional datasets including single-cell RNA-seq (scRNA-seq) and microscopy, and then compared the results to the deconvolution table F and to unsupervised decomposition (Figure 2A, schema).*

Major comment 5c. Significance and outliers in Figure 2C
Reviewer comment Figure 2C needs to show P-values and explain outliers in the text.
*Author response We have annotated the figure to state how each comparison is not significant and added additional text describe how this is related to high variability between samples.
Excerpt from Results, Evaluation of deconvolution results by scRNA-seq, page 5 In aggregate, the asthmatic group had fewer B-cells and more myeloid cells (Fisher’s Exact test, p-values 2e-8 and 7e-7 for B-cells and myeloid cells, respectively). However, the number of cells in each cluster was highly variable between individuals; for example, nearly all of the B-cell cluster was found in one individual (Figure 2C, Control B-cell outlier). For these five patients, the number of cells in the myeloid lineage cluster showed the most distinct separation between asthmatics and controls, but was not significant (Mann-Whitney-Wilcoxon signed rank test, p-value = 0.09) (Figure 2C). From this analysis, we concluded that (1) the blood-derived cell profiles appropriately fit the sputum cell types, (2) no additional cell types were needed to deconvolve the sputum bulk RNA-seq data, and (3) the high variability would require larger sample sizes to detect differences between asthmatics and controls.*

Major comment 5d. Selection of samples for microscopy
Reviewer comment Figure 2D - In the Results, the number of samples and how these were selected need to be explained.
*Author response We have added text to the results to describe the selection process.
Excerpt from Results, Evaluation of deconvolution results by microscopy, page 5 Second, we evaluated a random subset (~50%) of the samples by microscopy and manually counted the number of neutrophils, eosinophils, lymphocytes, and macrophages.*

Major comment 5e. Description of LDA topic membership
Reviewer comment Figure 2E - What are key elements of the topics? A Supplementary Table with the topic composition as well as some description in the text is necessary.
*Author response We have increased the description of several topics in the text and included a visualization of the top genes and a supplemental table of the full membership.
Excerpt from Results, Evaluation of deconvolution results by unsupervised decomposition, page 5 We correlated the cell-type fractions table with the gene topics fraction table (R(𝑭∙, 𝒇, 𝜽∙, 𝒌) for all patients, and found agreement between LDA and the cell-signature-based deconvolution for the most prominent cell type, neutrophils (p-value = 1.7e-34) (Figure 2D, topic 4). Gene-set enrichment analysis of the genes in this topic showed enrichment in the neutrophil chemotaxis pathway (Figure S8 B). The top genes in each topic can be found in Figure S7 and the complete membership in Table S3.*

Major comment 5f. Completeness of figure captions
Reviewer comment Figure 2F - Abbreviations should be explicit in the captions. This Figure is supposed to support the statement: "For example, the fraction of T-regulatory cells negatively correlated with the number of hospitalizations per year, suggesting a beneficial role of these cells in the management of asthma." What evidence or analysis suggests the T-reg faction predicts hospitalizations? Does an AUC curve show the relative weighting and strength of the topics in predicting particular clinical variables?
*Author response Explanations of each acronym is now provided in the figure legend.

Regarding the Treg sentence, the supporting data is a selection from the heatmap, specifically the significant negative correlation between the Treg relative abundances and the HPY (Hospitalizations Per Year). That is, in this cross-sectional analysis, individuals with fewer Tregs in their sputum had a medical history that included fewer hospitalizations. We have added text to the figure legend to clarify the method.
Excerpt from Figure legends, page 16. F) Pearson correlation of the Cell fraction table F and the Clinical table C. Only significant correlations after FDR correction are shown. ACT = Asthma Control Test score, Age.DX = age of asthma diagnosis, Age.SX.Onset = age of symptom onset, BDR = bronchodilator response, BMI = body mass index, FENO = forced expiratory nitric oxide, FEV1.FVC.postBD = the ratio of forced expriatory volume in one second to the forced vital capacity after treatment with a bronchodilator, FEV1.FVC.preBD = the ratio of forced expriatory volume in one second to the forced vital capacity before treatment with a bronchodilator, HIL = hospitalizations in lifetime, HPY = hospitalizations per year, ICS = average daily inhaled corticosteroid use, Number.of.OCS = average number of oral corticosteroids used, OCS.Total = lifetime total oral corticosteroid use.*

Major comment 6. Supplemental Table X
Reviewer comment 6. Page 7 Line 57 - Mentions "Supplementary Table X" which is not in the submission.
*Author response These count tables are included in the submission to dbGaP and will be made available at BioProject SUB7102729. Reference to the table has been removed.
Excerpt Results, Analysis of exogenous reads, page 6 The exogenous sequences aligned to mostly bacteria and fungi, although we also observed a few arthropod and helminth reads. The dominant phyla observed were from the bacterial kingdom: Proteobacteria, Firmicutes, and then Bacteroidetes.*

Major comment 7. Supplemental Figure Z
Reviewer comment Page 8 Line 43 - Mentions "Supplementary Figure Z" which is also not in the submission.
*Author response This figure has been re-labeled as Figure S5 and is included in the supplemental figures.
Excerpt from page 7: Similarly, we could denoise the microbe table using a co-abundance network, by correlating the genus-level abundances (R(𝑴∙, 𝒎, 𝑴∙, 𝒎) and identifying significant modules (Figure S5).*

Major comment 8. Significance of correlations and their relationship to causality
Reviewer comment 8. Page 8. Line 7 - What is the statistical significance of the correlations? An important caveat for the whole manuscript is that correlation does not imply causation. How would the authors translate their correlations into useful insights into disease biomarkers, diagnostics or therapeutics?
*Author response P-values that have been corrected for multiple-hypothesis testing are now defined for each correlation mentioned in the text.

Regarding causation, this study is cross-sectional and therefore unable to speak directly to causal roles. We include a comment to this effect in the Discussion.
Excerpt from Discussion, page 9 While experimental validation of these links is needed, these results represent observations that would be missed by analyses that do not effectively de-noise RNA-seq data in a way that maintains the interpretability of cell types, or that analyze only human RNA-seq reads. Additional follow-up is needed to establish a causal connection between these correlations.*

Major comment 9. Discussion-like sentences in the Results section
Reviewer comment 9. Page 8 and throughout. There is an ad hoc mixture of Results and Discussion sections in the manuscript. The manuscript needs to be restructure with a factual Results section, then relating those findings to the existing literature in the Discussion.
*Author response The results and discussion have been more clearly delineated as described. Many of the sentences in the results that related the observations to previous findings were aggregated into a new paragraph in the discussion, below.
Excerpt from Discussion, page 9. While several links imply that the presence of a microbe has triggered an inflammatory response, other interactions are also suggested. The gene lactotransferrin was linked to Aeromonas, which has been associated with gastroenteritis and skin infections and has been previously reported to bind lactoferrin [30]. Burkholderia, a gram-negative bacterial genus, is recognized as an important pathogen in the mucus-filled lungs of patients with cystic fibrosis; it was linked to gene MUC6, which encodes a secreted protein responsible for the production of mucin [31]. Classical pathogenic-type interactions included Haemophilus linked to NFKB Inhibitor Zeta, which is induced by the bacterial cell wall component lipopolysaccharide [32]. In addition, Haemophilus was linked to the cytokine interleukin 1 beta (IL1B), an important mediator of the inflammatory response. IL1B hypersensitivity is a hallmark of the asthma phenotype. Pasteurella was also linked to IL1B, and its toxin has been shown to induce expression of IL1B [33]. [18][19]*

Major comment 10. Definition of asthma severity
Reviewer comment Page 8 Line 35 - What is the clinical measurement of "asthma severity"? It is likely a multi-factorial index of patient wellness and lung function.
*Author response A brief description of the “asthma severity” label has been added to the text, with a more thorough description in the supplementary text related to the cohort.
Excerpt from Results, Dimensionality reduction for microbes: clustering and networks, page 6-7. The hierarchical clustering showed that the phylum distributions formed three clusters of patients. We related these clusters to the clinical variable “asthma severity”, defined by the amount of fluticasone or equivalent per day used to control symptoms (mild = <200 μg, moderate = 200-800 μg, severe = >800 μg).*

Major comment 11. Novelty of finding
Reviewer comment Page 8 Line 37 - The observation that lung Proteobacteria is associated with asthma or only lung diseases, such as COPD, is not novel (see https://www.ncbi.nlm.nih.gov/pubmed/31612122).
 *Author response We agree, and have highlighted this and a number of other previously reported results to give greater confidence in the method. We have added text to better define our logic and how we see reproducing previously reported results in the context of a sputum RNAseq dataset as adding value. In addition, we feel this gives greater confidence in the novel findings, which we have more clearly separated from those previously reported.
Excerpt from Introduction, Linking high-dimensional, heterogeneous datasets, page 2
Moreover, generating RNA-seq data from sputum can be technically easier than other methods to study microbe and immune cell interactions, such as by culturing sputum microbes or by flow-sorting sputum immune cells.*

Major comment 12. Accuracy of summary of current state of the literature
Reviewer comment 12. Page 8 Line 53 - The statement "How much cross-talk exists between microbes and human cells in the airway remains contentious [17]" is disingenuous. There are many papers linking bacterial metabolites to modulation of human immunity (for a recent review, see:
https://www.ncbi.nlm.nih.gov/pubmed/31501577 ).

*Author response We appreciate that there is strong and increasing support for the gut-lung axis whereby microbial metabolites affect respiration, as described in the review article. However, regarding the airway microbiota, we agree with the review article’s statement, “Further study of the lung ecosystem is needed to ascertain the impact of lung colonization on host physiology.” (Wypych et al 2019).

We have edited the sentence to clarify that we are referring to the microbes in the airway interacting with the cells in the airway, not the gut-lung axis.
Excerpt from Results, LDA-link for the identification of links between genes and microbes, page 7. How much cross-talk exists between the airway microbes and human cells remains contentious [15]. We feel this is partly due to the heterogeneous and noisy data from airway samples, where it is often difficult to find strong correlations using standard algorithms. We therefore sought to link genes to microbes via a method we dubbed LDA-link.*

Major comment 13. LDA-Link training and cross-validation
Reviewer comment Page 8 Line 53-61 & Page 9 Line 1-29. The LDA-Link section is highly flawed because its training set is a subset of the existing study data built on the same LDA associations. A true training set should be from a different data-set. Also, there is no mentioned of cross-validation of the model.
*Author response We appreciate this comment and have added text in the Results and Methods and a supplemental figure to more clearly describe the method. As was pointed out, the most challenging part of this modeling framework is the lack of gold-standard labels for the data, or a ground-truth data-set available for training. This required a self-supervised learning approach, which has been applied to many areas such as computer vision, machine learning and natural language processing(NLP) \cite{Jayaraman et al 2016, Pathak et al 2016, Raina et al 2007, Pathak et al 2017, Lynch et al 2019, and Wu et al 2019}. A variety of methods have been developed to infer the labels, such as an unsupervised representation (features that are very tightly linked together by some metric are assigned to a cluster and are given a label), intuitive knowledge transfer and integration \cite{Doersch et al 2015} and others \cite{Lynch et al 2019, and Wu et al 2019, Kong et al 2013}, etc.
The method we felt most appropriate is a form of the unsupervised representation method described above, where our clustering metric was a strong linear correlation. We sought to validate this method by gene-set enrichment analysis and found the pseudo-gold standard genes to be enriched in asthma and host-microbe interactions. We then used these labels to bootstrap a supervised approach for the remaining unlabeled data following standards in other fields described above.
Model accuracy was assessed using k-fold cross-validation, with a strict leave-out procedure to avoid partial overlap between the training and test sets. Supplemental figure S11 describes the process.
Both genes and microbes can make many to many connections (i.e. a gene can link to multiple microbes, and vice versa). To avoid information leakage between the training and test sets, the genes and microbes in the test set were excluded from training.

References:
Dinesh Jayaraman, Kristen Grauman. Learning image representations tied to egomotion from unlabeled video. 2016
Deepak Pathak, Philipp Krahenbuhl, Jeff Donahue, Trevor Darrell, Alexei A. Efros. Context Encoders: Feature Learning by Inpainting. 2016
Raina, Rajat and Battle, Alexis and Lee, Honglak and Packer, Benjamin and Ng, Andrew Y. Self-taught Learning: Transfer Learning from Unlabeled Data. ICML 2007
Bengio, Yoshua and Courville, Aaron and Vincent, Pascal. Representation Learning: A Review and New Perspectives. TPAMI 2013.
Deepak Pathak, Pulkit Agrawal, Alexei A. Efros, and Trevor Darrell. Curiosity-driven Exploration by Self-supervised Prediction. ICML 2017
Corey Lynch, Mohi Khansari, Ted Xiao, Vikash Kumar, Jonathan Tompson, Sergey Levine, Pierre Sermanet. Learning Latent Plans from Play. 2019
Jiawei Wu, Xin Wang, William Yang Wang. Self-Supervised Dialogue Learning. ACL 2019
Kong et al Inferring Anchor Links across Multiple Heterogeneous Social Networks. 2013

Excerpt from Results, LDA-link for the identification of links between genes and microbes, page 7

Methods, Pathogen-to-host correlation and link identification, pages 13-14

As is often the case, these RNA-seq data lack a gold standard set of labels for the links between genes and microbes that can train a machine learning algorithm. Therefore, LDA-link uses a strategy similar to self-supervised methods, which create a pseudo-gold standard, train a supervised model using the pseudo-gold standard, and then apply the model to the remainder of the data [22-29]. In this case, the training set was defined by a small number of very strong linear correlations and an equal number of very low correlations.

We employed a self-supervised learning framework to learn labels from the data. The links between gene and microbes that were very tightly correlated, ergo they were in one cluster, were labeled as the positive links after being confirmed by functional enrichment (gene-microbe correlations with p-values less than 1e-5 (FDR = 0.016 and absolute correlation greater than 0.4)). Negative links in the training set were defined as an absolute correlation of less than R(i,j) = 0.05. Each gene and microbe could be linked to many microbes or genes, respectively.

Cross-validation was performed using a strict left-out process, as shown in Figure S11. Ten percent of the training data (true-positive and negative labels) were separated as the test set, and any microbes and genes in this test set were removed from the remaining training data, leaving slightly less than 90% of the pseudo-gold standard for training. A randomForest model was trained on the remaining labels, and then tested on the left-out 10%. We repeated these procedures 10 times to estimate receiver operator characteristics of the model; the area under the curve was approximately 0.7.*

Major comment 14. Known mechanisms of interaction between bacteria and human cells
Reviewer comment Page 9 line 33-50 - This review of previous work does not clearly different between human receptor cross-talk with the microbiota versus innate immunity or auto-immunity response to pathogens. This should be in the Discussion as well. However, there is still a question about the novelty of the LDA approaches given that the uncovered relationships are known.
*Author response We agree with the reviewer and have added a sentence to the introduction to better represent the divsersity of interactions that are possible in this context.

However, we hesitate to discuss the biology in significant detail in this “Method” article. In the discussion we chose instead to further clarify the value in identifying known relationships from this type of dataset. More specifically, we feel it appropriate to highlight when our new method identified known and established links between genes and microbes in order to increase the confidence in the novel findings.
Excerpt from Introduction, Interactions between the host and microbes in the lung, page 2

Discussion, page 9
The majority of the organisms in the lungs are expected to have no effect, and severe asthmatics are expected to have more pathogenic and fewer beneficial microbes. However, the reality is more complex; organisms can interact with the lung in myriad ways, such as through innate responses to pathogens, small-molecule interactions and others [2, 3].

A number of the links identified here have been described previously through different experimental methods. For example, a severe form of asthma has been characterized by eosinophilia [44], and pulmonary candidiasis has long been associated with allergic bronchial asthma and inflammation [45]. However, the methods used to uncover these relationships we argue are more challenging than total RNA-seq of sputum or provide less information. For example, asthmatic eosinophilia was identified by microscopic counting of cells [44], as we used to validate deconvolved cell counts (Figure 2D). In the case of candidiasis, the observation was made by culturing [45]. [18][19] ...

Having gained confidence in the method by finding previously-reported associations we then turn to the novel findings. The links identified here suggest processes by which lung immune cells respond to microbes. We found that mast cells respond to Haemophilus and Pasteurella via IL1B and that eosinophils respond to Candida via GCSAML....*

Major comment 15. Accuracy of summary of current knowledge
Reviewer comment Page 10 Line 29-35. The statement, "A limited number of reports have investigated the changes in the lung microbiota between healthy, non-smoking and smoking individuals as well as in patients suffering from cystic fibrosis, chronic obstructive pulmonary disease, or 317 asthma [2, 28-30]. Despite emerging data on the airway microbiota, little is known about the role of the lung microbiome in modulating pulmonary mucosal immune responses…" is inaccurate. A search of PubMed using the term "lung microbiome asthma" retrieved 260 citations. For the "lung microbiome" alone, there are 1480 citations.
*Author response We agree have updated the text to describe the current state of the literature. It was our intention to relate the amount known about the lung microbiota to the amount known about the gut and have added text to clarify this point.
Excerpt from Discussion, page 9. Investigating the microbiota in the lower respiratory tract is a relatively new field in comparison to the extensive work on the intestinal tract. In fact, the lung was excluded from the original Human Microbiome Project because it was not thought to have a stable resident microbiome [17]. Since this initial lag, reports have defined commensal microbes within healthy individuals and identified consistently enriched or depleted taxa in such conditions as asthma, chronic obstructive pulmonary disease, and cystic fibrosis, to name a few [4, 40-43]. LDA-link facilitates the observation of relationships between microbes and genes and link them to immune cells and their responses. [2, 26-28]*

**Second round of review**

**Reviewer 1**

The authors adequately addressed my questions and concerns.

**Reviewer 2**

The authors went to considerable effort to address comments in my initial review. I believe that the current revised manuscript warrants publication.
